# Modeling somatic and dendritic spike mediated plasticity at the single neuron and network level

**DOI:** 10.1038/s41467-017-00740-z

**Published:** 2017-09-26

**Authors:** Jacopo Bono, Claudia Clopath

**Affiliations:** Department of Bioengineering, Imperial College London, South Kensington Campus, London, SW7 2AZ UK

## Abstract

Synaptic plasticity is thought to be the principal neuronal mechanism underlying learning. Models of plastic networks typically combine point neurons with spike-timing-dependent plasticity (STDP) as the learning rule. However, a point neuron does not capture the local non-linear processing of synaptic inputs allowed for by dendrites. Furthermore, experimental evidence suggests that STDP is not the only learning rule available to neurons. By implementing biophysically realistic neuron models, we study how dendrites enable multiple synaptic plasticity mechanisms to coexist in a single cell. In these models, we compare the conditions for STDP and for synaptic strengthening by local dendritic spikes. We also explore how the connectivity between two cells is affected by these plasticity rules and by different synaptic distributions. Finally, we show that how memory retention during associative learning can be prolonged in networks of neurons by including dendrites.

## Abstract (web summary)

Synaptic plasticity is the neuronal mechanism underlying learning. Here the authors construct biophysical models of pyramidal neurons that reproduce observed plasticity gradients along the dendrite and show that dendritic spike dependent LTP which is predominant in distal sections can prolong memory retention.

## Introduction

Our brains continuously process novel information and provide us with remarkable flexibility to adapt and learn in an ever-changing environment. Understanding which mechanisms underlie this flexibility is an important step towards understanding how sensory stimuli are processed in cortical areas. A seminal idea, now widely known as Hebb’s postulate, suggested that synapses are the neurological substrates for learning^1, 2^. In short, Hebb proposed that a neuron A persistently taking part in the firing of a neuron B leads to increased synaptic efficacy from A to B. Experiments later showed that high-frequency stimulation indeed evoked persistent increased efficacy, termed long-term potentiation (LTP), at hippocampal synapses^3^. Long-term depression (LTD), predicted as a mechanism to balance LTP^4, 5^, was later shown to be evoked by low-frequency stimulation^6^.

Subsequent discoveries showed how the precise timing between the presynaptic and postsynaptic activity influences plasticity. When the presynaptic neuron fires just before the postsynaptic neuron, the synapse is potentiated; reversing the firing order leads to synaptic depression^7–9^. This form of plasticity, where the precise timing of spikes determines the subsequent synaptic change, is called spike-timing-dependent plasticity (STDP), and is a widely used learning rule in computational studies^10–12^. Furthermore, the synaptic changes have been shown to be also dependent on postsynaptic depolarisation^13–15^, triples and higher order multiples of spikes^16–18^, the dendritic location of the synapse^19–22^ and on the rate of presynaptic and postsynaptic activity^8, 23^. These results have led to refined models for synaptic plasticity, among which are mechanistic models based on the calcium hypothesis^24, 25^, triplet models^26^ and a voltage-dependent phenomenological model^27^, which we use in this study.

STDP requires the generation of somatic sodium (Na^+^) spikes and their back-propagation into the dendrites. These two requirements limit the capacity of STDP to explain a number of observed plasticity phenomena. First, pair-based STDP cannot account for activity-dependent learning with weak inputs, which are not powerful enough to evoke action potentials. Second, learning in dendritic regions far from the soma is problematic due to the attenuation or failure of the back-propagating action potential (bAP)^19–21^. Finally, an increasing number of experimental studies have revealed plasticity mechanisms that do not rely on postsynaptic action potential generation, but instead on local postsynaptic dendritic spikes^28–34^ or sub-threshold events for dendritic spikes^35, 36^. The requirement for somatic action potentials for the induction of synaptic changes has therefore been disputed^37–43^.

In this article, we investigate how dendrites allow for multiple plasticity rules to be present in a single cell, and attempt to compare their relative importance. In biophysical models of a cortical layer 5 and layer 2/3 pyramidal neuron, we implement the voltage-dependent plasticity rule^27^, which reproduces the various plasticity phenomena described above^8, 13–23, 28, 31^. Focusing on the basal dendritic tree, our simulations reproduce experimental evidence of a gradient of plasticity along these dendrites, with STDP dominating proximally and a dendritic spike-dependent LTP (dLTP) distally. We then investigate how synaptic distribution affects the connectivity between two neurons and how NMDA spikes can determine the direction of plasticity at other synapses. Finally, we explore the influence of dLTP in the context of associative learning in single cells and networks of simplified neurons. In particular, we find that dLTP can prolong memory retention.

## Results

**Fig. 1:**
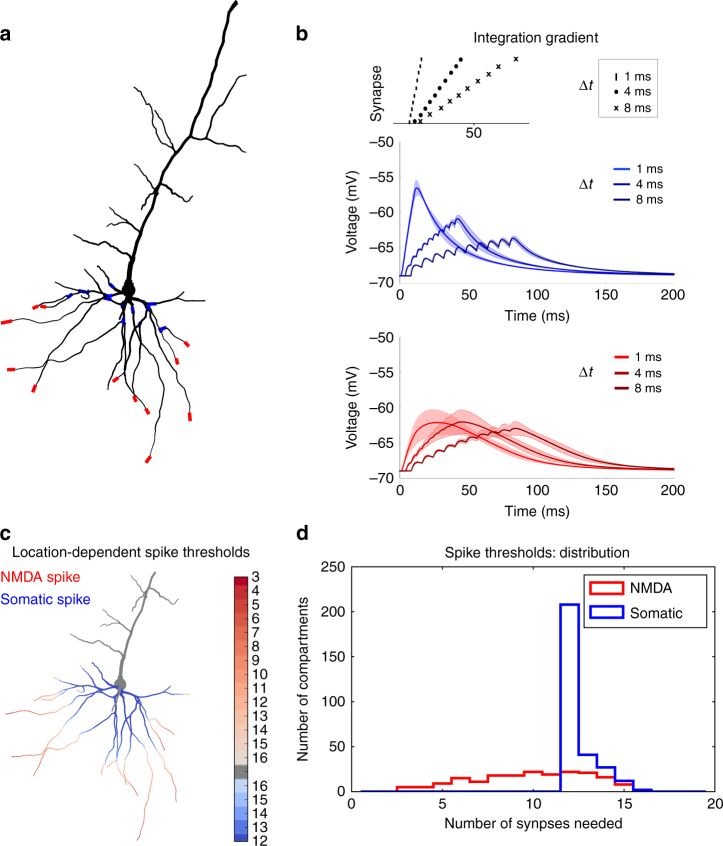
Location dependence of local and global regenerative events. **a** Proximal (*blue*) and distal (*red*) locations on a thin basal branch of the detailed neuron model. **b** Temporal integration gradient along a basal dendrite (reproducing experimental results by Branco and Häusser^47^). *Top panel*: activation sequences with different interspike intervals. Middle panel: proximal stimulation requires high temporal coincidence, as the somatic depolarisation drops substantially when inputs are not active synchronously. The solid line is the mean over all proximal compartments shown in panel **a**, the shaded region is one s.d. *Bottom panel*: distal stimulation results in similar somatic depolarisation over a bigger range of interspike intervals. The solid line is the mean over all distal compartments shown in panel **a**, the shaded region is one s.d. **c** For each dendritic compartment in the neuron model, synapses are activated until either an NMDA spike or an action potential is generated. Stimulating proximal compartments results in an action potential before reaching the threshold for NMDA spikes (*blue*). Stimulating distal regions results in NMDA spike generation while staying sub-threshold for somatic spikes (*red*). Intenser colours denote that less synapses are needed to evoke a spike. The numbers next to the colour bar represent the number of synapses needed for a spike. **d** For each compartment in **c**, the number of synapses needed to evoke the spikes was stored and represented as a histogram. NMDA spikes (*red*) generally require less synapses in our model than somatic spikes (*blue*)

**Fig. 2:**
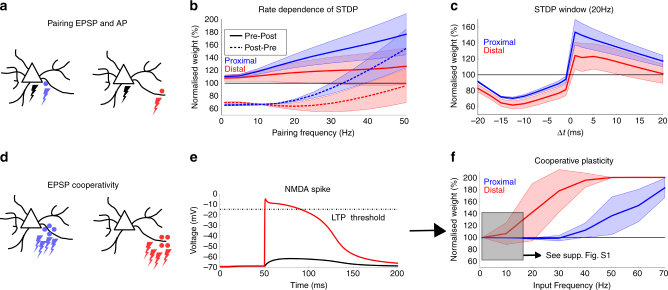
Plasticity gradient along basal dendrites. **a** Action potentials (*black lightning*) are paired with either proximal (*blue*) or distal (*red*) synaptic activations. **b** The pairing protocol as in Sjöström et al.^23^ is simulated. Action potentials and EPSPs are paired with a fixed interval of 10 ms, either pre–post (*full lines*) or post–pre (*dashed lines*). Five pairings at a fixed frequency are repeated 15 times (Methods section). The protocol is repeated for several pairing frequencies (*horizontal axis*) and the weight is displayed normalised to the initial value (*vertical axis*). The plasticity parameters were chosen to qualitatively reproduce the experimentally observed rate-dependence. Distal synapses show less potentiation due to the attenuation of the bAP. The solid lines are the means over all proximal (*blue*) or distal (*red*) compartments shown in Fig. 1a, the shaded regions are one s.d. **c** The same protocol as in **b** is repeated at a pairing frequency of 20 Hz, but the interspike interval is now varied (*horizontal axis*). The weight is normalised to the initial value (*vertical axis*) and shows the standard STDP window, where the potentiation at distal synapses is reduced compared to proximal synapses due to the attenuation of the bAP. The solid lines are the means over all proximal (*blue*) or distal (*red*) compartments shown in Fig. 1a, the shaded regions are one s.d. **d** A group of synapses on a proximal compartment (*blue*) or distal compartment (*red*) are activated by a Poisson process. **e** Distal groups of synapses can elicit NMDA spikes (*red trace*), which cross the LTP threshold of the plasticity model and can therefore result in potentiation while remaining sub-threshold in the soma (*black trace*). **f** When activating the synaptic groups as described in **d**, LTP is observed at much lower average activation rates for the distal group (*red trace*) compared to the proximal group (*blue trace*). This dLTP compensates for the lack of potentiation by STDP **b**, **c**. The solid lines are the means over all proximal (*blue*) or distal (*red*) compartments shown in Fig. 1a, the shaded regions are one s.d. Supplementary Fig. 1 shows distal depression at low rates (*black shaded region*) more clearly

We modelled both biophysically realistic neurons (one cortical layer 5 (Fig. 1a) and one layer 2/3 (Supplementary Fig. 3a) pyramidal neuron) as well as simplified neurons (Supplementary Fig. 5). The reconstructed morphologies of the biophysical neurons were taken from ref. ^44^ and ref. ^45^, respectively. We observe similar results for the layer 5 model (Figs 1 and 2) as for the layer 2/3 model (Supplementary Figs 3 and 4). In our morphologically reconstructed neurons, we added Na^+^, K^+^ and Ca^2+^ conductances based on the fit to experimental data described previously in Acker & Antic^44^ (see Methods). In the simplified neuron model, each dendrite consists of two point-like compartments; the somatic compartment is modelled as an exponential integrate-and-fire unit. The somatic and dendritic compartments are electrically coupled and the coupling strengths are chosen to reproduce the behaviour of the somatic, proximal and distal regions in the biophysical model (Supplementary Fig. 5). The synapses in both models have α-amino-3-hydroxy-5-methyl-4-isoxazolepropionic acid (AMPA) receptors and N-methyl-D-aspartate (NMDA) receptors, but only the AMPA components are plastic under a voltage-dependent plasticity rule^27^. Similar results are obtained when both AMPA and NMDA conductances are plastic (Supplementary Figs 6–10). All simulations of the biophysical neurons were performed using Python with the Brian2 simulator^46^, the network simulations were performed using Python. The code for both models and all simulations herein is available on ModelDB (https://senselab.med.yale.edu/modeldb/).

To avoid further ambiguities, in light of inconsistencies in the current literature, we point out to the reader that by distal, we denote the regions furthest from the soma on basal dendrites, as opposed to the apical tuft dendrites; and by proximal, we mean the regions closer to the soma on basal dendrites, as opposed to the whole basal dendritic tree.

### Location dependence of local and global regenerative events

We focus on two types of regenerative events that lead to synaptic changes, somatic sodium spikes and dendritic NMDA spikes. To understand how these events contribute to synaptic plasticity, we need to understand where in the dendritic tree, and by which synaptic activities, they are evoked. Therefore, we, first turn our attention to the locations of global sodium spikes and of local NMDA spikes, and the inputs required to elicit them.

Our biophysical model was able to reproduce the observed integration gradient along thin basal dendrites, previously reported in both experimentally and in a computational model^47^ (Fig. 1a, b). This gradient arises from a difference in input impedance along the thin dendrites, resulting in greater depolarisation by single excitatory postsynaptic potentials (EPSPs) at distal locations on the dendrite compared to proximal locations. In turn, the higher depolarisation allows more NMDA-receptor mediated channels to open; this voltage-dependence ensures that sufficiently strong synaptic input can evoke a local dendritic NMDA spike^30, 48–50^.

We then quantified the NMDA-dependent integration gradient in our biophysical model in more detail. For each of the dendritic compartments, we recorded the minimum number of synapses required to induce either a somatic spike or an NMDA spike. In our model, synapses clustered on the proximal dendritic compartments need less input to elicit somatic spikes than NMDA spikes, while for synapses clustered on distal dendritic compartments, less input is required to evoke an NMDA spike (Fig. 1c). Moreover, the number of synapses needed to generate an NMDA spike is generally much lower than that for a somatic spike (Fig. 1d). While the latter simulation was performed with a 1:1 AMPA to NMDA ratio, the results remain valid over a range of biologically plausible AMPA to NMDA ratios^51^ (Supplementary Fig. 2).

Our model also confirms that increasing the synaptic input to a distal compartment usually does not result in a combination of NMDA spikes and somatic spikes. Increasing the input to such a compartment will increase the duration, but not the amplitude, of the plateau, resulting in a longer, but still sub-threshold, signal at the soma^48^. However, combining such a plateau depolarisation with inputs from different compartments might evoke somatic spikes (Fig. 4).

### Location dependence of STDP and dLTP

To investigate how the integration gradient described in the previous section affects synaptic plasticity, a local voltage-dependent plasticity rule was implemented in our model. The plasticity rule is based on previous work from Clopath et al.^27^ and the parameters were re-fitted to reproduce the depolarisation- and rate-dependence of plasticity^8, 13–15, 23^ (Fig. 2b). Our model is consistent with experimental results reporting a reduced potentiation window at further distances from the soma, due to the attenuation of the back-propagating action potential (bAP)^19–21^ (Fig. 2c). We also found that the same plasticity rule was able to account for potentiation dependent on local dendritic spikes, i.e. dLTP, observed both in vitro^28–30, 32, 35^ and in vivo^31, 34^. Because the plasticity rule depends on voltage rather than postsynaptic spiking, a large postsynaptic depolarisation caused by a dendritic spike is sufficient for LTP and no back-propagating action potential (bAP) is required (Fig. 2e).

Since NMDA spikes are more easily evoked in the distal regions of a dendrite (Fig. 1c, d), we investigated whether dLTP, without the need for action potentials, is more easily evoked at the distal locations. For this purpose, we stimulated 10 synapses placed either distally or proximally on the basal dendrites using a Poisson process, and repeated this stimulation with different average activation rates. The synaptic groups located distally led to potentiation at substantially lower activation rates, compared to the proximal groups (Fig. 2f).

Our simulations are consistent with the experimental observation of a plasticity gradient along basal dendrites. STDP is more effective in proximal regions, while dLTP is more easily evoked at distal regions. The latter mechanism could rescue the distally reduced potentiation caused by an attenuated bAP. Note that, so far, we have only discussed an additional mechanism for LTP at distal locations, evoked by NMDA spikes. However, depression at distal locations can also occur simply by inputs that remain sub-threshold for NMDA spikes. This can be achieved by low-rate activation of distal synapses (Supplementary Fig. 1a), or by simultaneous inhibitory input (Supplementary Fig. 1b–e).

### Synapse distribution influences connectivity

**Fig. 3:**
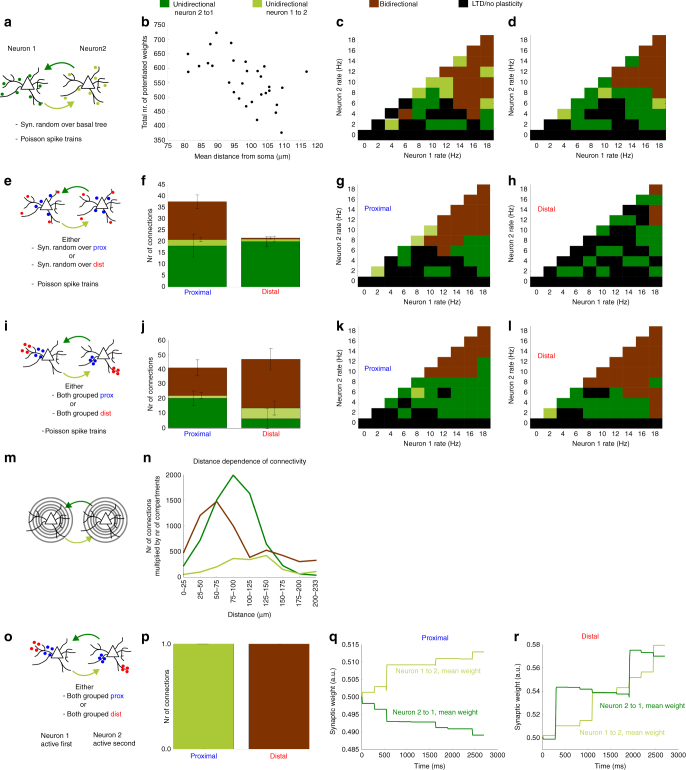
Predicted connectivity along dendrites. **a** Two neurons are mutually connected: the synapses are randomly distributed across the basal dendrites and both neurons are activated by generating Poisson spike trains and injecting a brief external current into the somatic compartments at the relevant spike times. **b** The mean distance of the synapses to the soma is negatively correlated to the number of strengthened synapses (Pearson’s *r* = −0.569, *p* < 2e−3). **c**, **d** Two examples of the final connectivity. Colour codes can be found at the legend on top of the figure. **e** Same as panel **a**, but the synapses are now constraint to either proximal or distal compartments. **f** More synapses are strengthened when targeting proximal regions compared to when targeting distal regions. The stacked bars show the means over five simulations (*error bars* indicate one s.d.). **g**, **h** Examples of final connectivity for the proximally distributed case **g** and the distally distributed case **h**. **i** Analogous to panel **e**, but synapses are clustered onto one proximal or distal compartment. **j** Analogous to panel **f**, but synapses are now clustered onto one proximal or distal compartment. By comparing with **f**, proximal synapses are less influenced by the clustering than distal synapses. The *stacked bars* show the means over five simulations (*error bars* indicate one s.d.). **k**, **l** Examples of the final connectivity for the proximally clustered case **k** and the distally clustered case **l**. **m** We divide our basal dendritic tree in nine regions depending on the distance from the soma. For each distance range, synapses are either randomly distributed among the relevant compartments, or clustered on one of the compartments. **n** The mean results of connectivities for the distributed and the clustered simulations in each distance range are summed. The values are multiplied by the number of compartments in the respective distance range. **o** Neuron 1 is always activated before neuron 2. The synapses are clustered either proximally or distally. **p** In the proximal case, all simulations result in unidirectional connections from neuron 1 to neuron 2. In the distal case, only bidirectional connections are observed. **q**, **r** Examples of the evolution of the mean synaptic weights during the simulation in the proximal case **q** and the distal case **r**

We wished to explore how the plasticity mechanisms described in the previous section influence the connectivity between two neurons. To this end, we simulated two biophysical neurons that are mutually connected, and varied the distribution of synapses in the following ways: they are either randomly chosen across the basal tree (Fig. 3a–d); randomly distributed across only proximal compartments or only distal compartments (Fig. 3e–h); or grouped onto a single proximal or a single distal compartment (Fig. 3i–l, o–r). Furthermore, we activated the neurons by either generating Poisson spike trains over a range of rates (Fig. 3a–n), or by always activating one neuron before the second (Fig. 3o–r). The spikes were evoked by a brief external current injection into the somatic compartment, and not by synaptic activations (Methods section).

We then investigated the synaptic changes caused by these different synaptic distributions and stimulation protocols. First, we randomly distributed 15 synapses across the basal dendrites (Fig. 3a) while evoking Poisson spike trains in both neurons. For firing rates lower than 12 Hz, we observed mainly synaptic depression or unidirectional connections from the neuron with lowest firing rate towards the neuron with higher firing rate. For higher rates, bidirectional connections were formed (Fig. 3b, c). Since we were interested in whether the distance from the soma affected the development of strong connections, we calculated the average distance of synapses from the soma in each simulation and plotted this as a function of the total number of potentiated synapses (Fig. 3d). The data has a negative correlation coefficient (*r* = −0.569) suggesting that proximity to the soma favours LTP.

To further investigate this, we repeated the simulation, but with ten synapses distributed over either only the proximal or only the distal compartments (Fig. 3e, the compartments used for these simulations are the same as shown in Fig. 1a). The proximally connected neurons indeed clearly favoured potentiation compared to the distally connected neurons, and the resulting connectivity (unidirectional vs. bidirectional) depended on the neuronal firing rates (Fig. 3f–h). We can understand this result by noting that the synapses were randomly distributed in these simulations. Thus, the probability of finding clustered synapses is small, and STDP will dominate over dLTP. The bAP is therefore crucial for synaptic potentiation, and favours proximal synapses.

We then clustered all the synapses together onto one compartment, either proximally or distally (Fig. 3i). When the neurons are proximally connected in this way, the resulting synaptic changes are not substantially different from the case where the synapses are randomly distributed (compare Fig. 3j, k with Fig. 3f, g). However, at distal locations, an NMDA spike can easily be evoked by the clustered synapses; in turn, this changes the plasticity substantially (compare Fig. 3j, l with Fig. 3f, h). Even for the lowest firing rates, the collective activation of a distal synaptic group leads to an NMDA spike and therefore potentiation.

We next aimed to investigate the synaptic changes across a continuum of distances from the soma. We therefore divided our basal dendritic tree into nine different regions depending on the distance from the soma (Fig. 3m). For each distance range, we distributed synapses either randomly among the respective compartments, or they were clustered on one of the compartments in that range. The neurons were activated using Poisson spike trains with different average rates, and we stored the resulting number of unidirectional and bidirectional connections in each case. The mean of these numbers for the distributed and the clustered simulations are summed per distance range. While we ran an equal number of simulations for all distances, there were substantially fewer compartments for the smallest and largest distance ranges compared to intermediate ranges in our neuron. To compensate for this, we further multiplied the number of unidirectional and bidirectional connections by the number of compartments in the respective distance range (Fig. 3n).

Finally, while clustering the synapses on either a proximal or a distal compartment, we changed the activation pattern. Instead of generating Poisson spike trains simultaneously in both neurons, we added a temporal structure: the first neuron was always activated before the second (Fig. 3o). In this case, proximally connected neurons always become unidirectionally connected by both potentiating synapses from the first to the second neuron (pre–post activations), and depressing synapses from the second to the first (post–pre activations) (Fig. 3p, q). Conversely, distally connected neurons always form bidirectional connections, since every presynaptic activation generates an NMDA spike in the postsynaptic neuron (Fig. 3p, r).

We conclude that the location of synapses highly influences the connectivity between neurons. If synapses are not clustered, our model predicts that proximal connections can be strengthened even for relatively low rates (less than 10 Hz) while distal synapses only allow for LTP when the pre- or postsynaptic neuron fires at higher rates (10 Hz or more). However, if synapses are clustered on the same dendritic compartment, distal synapses lead to potentiation even at low firing rates and independently of any temporal order in neuronal activation. Therefore, we make the experimental prediction that observed pairs of neurons connected by distally clustered synapses should express mainly bidirectional connectivity, while pairs of neurons that do not show distal clusters should over-represent unidirectional connections. To test our predictions, we re-analysed published data from Markram et al.^52^. In this study, the average location of unidirectional connections is at 89.6μm ± 28.6 μm, whereas the average location of bidirectional connection is at 106.9μm ± 33.7 μm (see details in Methods section). These data are in qualitative agreement with the prediction made above.

### Plateau potentials can gate plasticity at other synapses

Dendritic NMDA spikes are powerful regenerative events, with long plateau depolarisations lasting up to hundreds of milliseconds^48, 49^. As they are more easily evoked at the terminal regions of basal dendrites, they undergo substantial attenuation and cause only sub-threshold events at the soma. This long sub-threshold plateau reduces the amount of depolarisation required to reach the spiking threshold and therefore enables other weak inputs to reach the threshold.

**Fig. 4:**
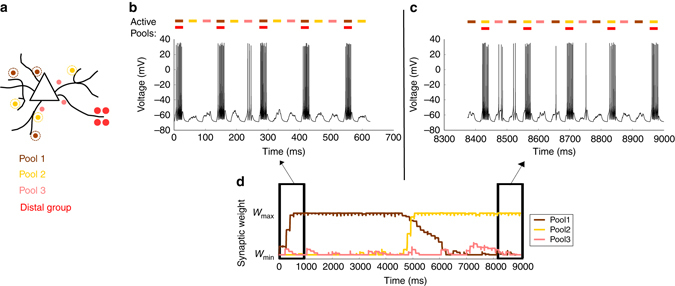
Distally evoked plateau potentials can switch the direction of plasticity at other synapses. **a** Different groups of synapses are connected to the neuron: three pools (*brown*, *yellow*, *pink*) are randomly distributed across the basal dendrites, and one group (*red*) is connected on one distal compartment. **b** The distal cluster (*red*) is paired with pool 1 (*brown*) in the first half of the simulation. The neuron almost exclusively reaches the spiking threshold when these synapses are active together. This leads to potentiation of the brown synapses (**d**, *first part*). **c** The distal cluster (*red*) is paired with pool 2 (*yellow*) in the second part of the simulation. The brown synapses depress again and the yellow synapses are potentiated (**d**, *second part*). As the distal (*red*) group evokes an NMDA spike every time it is activated, it remains at the maximum bound

We investigated how the interaction between plateau potentials and sub-threshold inputs affects plasticity. In particular, we explored the interplay between STDP and dLTP by connecting three pools of synapses randomly across the basal dendrites. One extra group of synapses is added at the distal part of a thin branch (Fig. 4a). The three pools are activated sequentially, but are too weak to induce potentiation by STDP. During the first half of the simulation, the activation of the first pool (Fig. 4, brown) is coincident with the activation of the distal group (Fig. 4, red). The weak inputs from the first pool paired with the plateau depolarisation now enable the neuron to elicit somatic spikes, which in turn lead to potentiation of synapses belonging to the first pool. During the second half of the simulation, the distal group is activated together with the second pool (Fig. 4, yellow) instead of the first. The synapses belonging to the first pool depress again, while those from the second pool, now coupled with the plateau potential, are potentiated (Fig. 4d).

Our computational model therefore suggests a potential computational role of these diverse plasticity rules, namely that distal inputs can “gate” which proximal inputs will learn.

### Retention of associations with dLTP in single cells

We now study the functional implications of dLTP. For this purpose, we consider a memory which associates several features. Such an association should be robust when presenting only its components, since neurons taking part in multiple assemblies and various sources of noise can lead to an incomplete activation of the association. For example, imagine that we have learned the association “coffee”, with components such as the colour brown, its taste, its smell, etc. We do not forget to associate the colour brown with the item “coffee”, even though we experience that colour much more often than we experience coffee. Since the number of synapses needed for an action potential is larger than that needed for an NMDA spike (Fig. 1d), we hypothesise that spatially clustered cooperating synapses allow for an increased retention of associations. Indeed, in the voltage-dependent plasticity rule, LTD depends on presynaptic activity and postsynaptic depolarisation (see Methods section). When a group of synapses is activated but fails to evoke a somatic or dendritic spike, the weights will be depressed. This situation can arise when activating only a fraction of the inputs, thereby leading to depression and, in turn, the unlearning or forgetting of the previously learned association. Therefore, we sought to determine how the different requirements to evoke somatic and NMDA spikes (Fig. 1) affect the retention of learned associations.

**Fig. 5:**
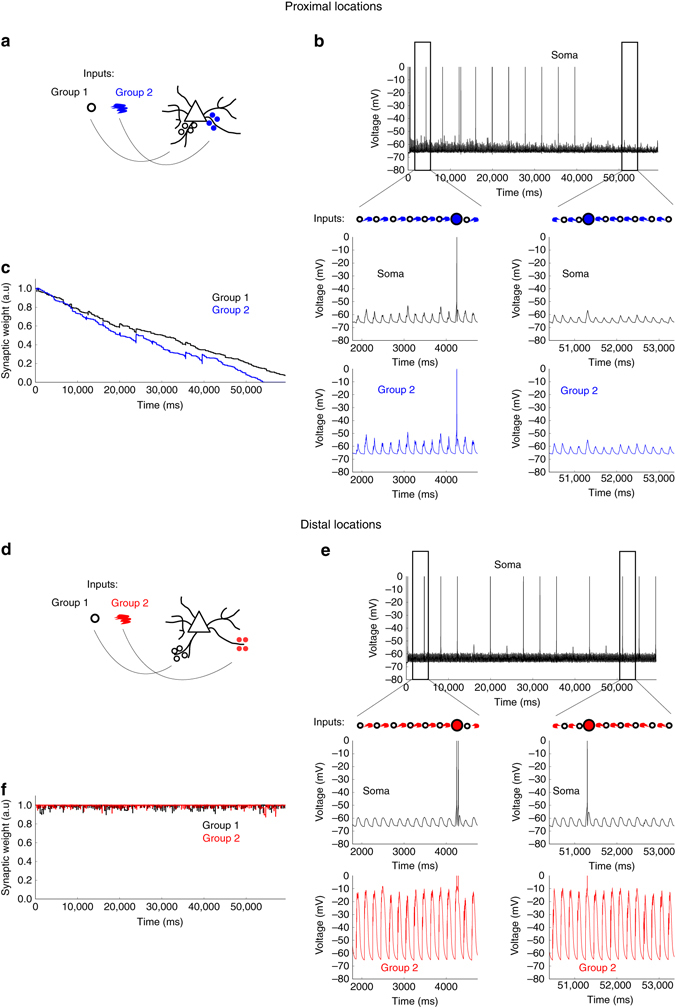
Sub-threshold inputs are maintained by dLTP. **a** Cartoon of the simulation: two groups of synapses are placed at proximal compartments. Group 1 codes for a circle, group 2 for the colour blue. While the groups are often activated separately, the neuron should only generate an output in the rare event of a blue circle (i.e., both groups active simultaneously). **b** Top panel: somatic trace during the simulation. Middle panels: detail of the somatic trace during the initial and final part of the simulation. While a blue circle leads to action potentials initially, it eventually becomes sub-threshold. Bottom panels: dendritic traces at the proximal location of cluster 2, corresponding to the somatic traces in the middle panel. **c** Evolution of synaptic weights of both groups during the simulation. The individual activations of the groups lead to depression, the simultaneous activations result in potentiation initially, but are insufficient to keep the synapses at the maximum weight. Finally, the simultaneous activations become sub-threshold and the weights depress towards the lower bound. **d**–**f** Analogous to **a**–**c**, but connecting the groups at distal locations on basal dendrites. The individual activations of the groups now lead to NMDA spikes (see panel **e**, bottom, for the second group), keeping the weights at the upper bound (**f**) and allowing the neuron to maintain its *red circle* representation (**e**, top and middle)

First, we activated two proximally grouped pools of neurons. We assumed that the neuron should respond only to the activation of both pools together, but not to the pools separately. For simplicity, the first pool is denoted as coding for circular shapes and the second one for the colour blue (Fig. 5a). We further assume that we encounter many non-blue circular shapes and many non-circular blue objects, while only sometimes observing blue circles. Hence, the separate activations of the circle-pool and the blue-pool will occur much more often than the simultaneous activation of both pools (blue circle) (Fig. 5b). Even though the simultaneous activations (blue circle) might elicit somatic spikes at the start of the simulation, the multitude of single-pool activations will depress the involved synapses, ensuring that even simultaneously activating the pools eventually becomes sub-threshold (Fig. 5b, c). This poses a fundamental limit on retaining cell assemblies with similar STDP models.

When two pools (this time denoting circular shapes and the colour red, Fig. 5d) are located at the distal parts on basal dendrites, each activation of a pool elicits an NMDA spike, therefore keeping the synapses at the maximum weight (Fig. 5e, f). Importantly, keeping the weights at full strength does not require any action potentials in this case, and the separate activations of each feature remain sub-threshold and do not interfere. This allows the neuron to reliably produce output in the event of a red circle only (Fig. 5e). Our example shows how dLTP provides a mechanism to maintain associations between various inputs by protecting the synapses from being overwritten by other activity. Underlying this behaviour is a crucial difference between dLTP and STDP. When using only STDP, the neuronal output also provides the learning signal for potentiation. Indeed, an action potential (output) is necessary for the induction of any weight change (learning). This is in contrast with dLTP, where the potentiation is completely decoupled from the output (action potential generation) but instead relies on NMDA spikes. Therefore, the learning can happen independently of the output, allowing the different features of the learned association to be activated separately without leading to depression.

### Memory retention in networks of neurons with dendrites

**Fig. 6:**
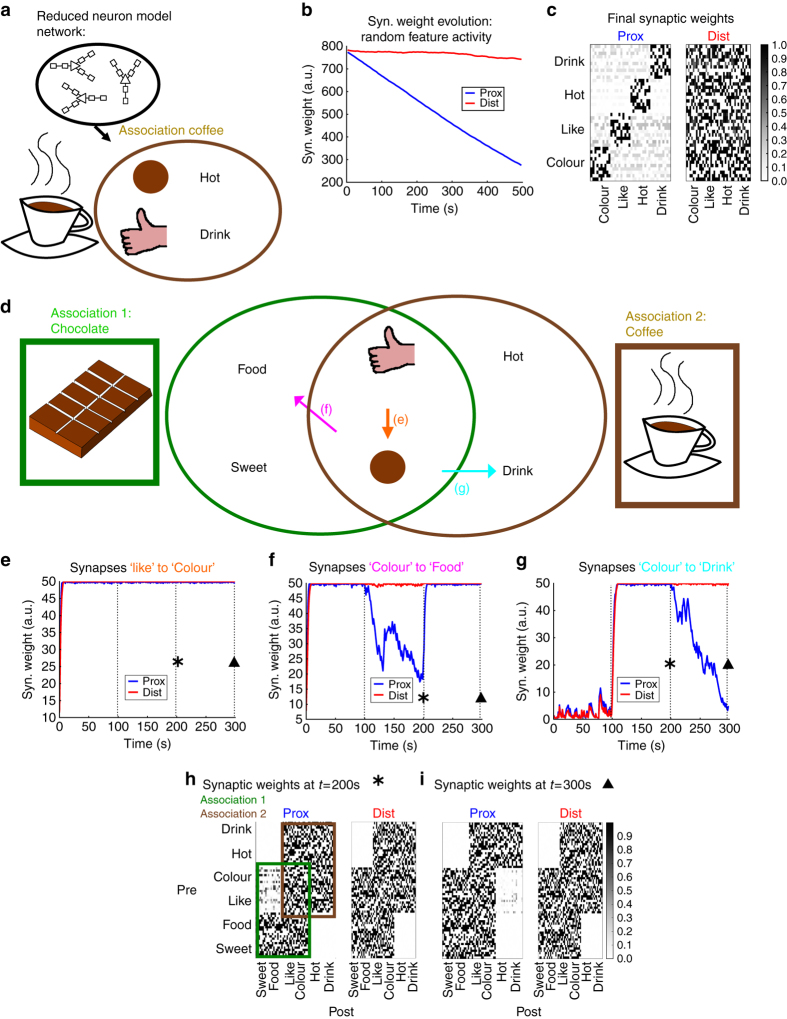
In networks, dLTP can protect previously learnt connections. **a** *Top*: A neuron model with simplified dendrites is implemented for the network simulations. The model consists of two compartments per dendrite, one representing a proximal compartment and one representing a distal compartment (see also Supplementary Fig. 5). *Bottom*: A network represents an associative memory (“coffee”), containing four subgroups of neurons which we will call “features”, in this case they are colour (brown), something you like, hot and drink. The initial weights are all-to-all and initiated at the maximum bound. **b** When randomly activating the individual features, the distal synaptic clusters are always activated together and evoke NMDA spikes. While proximal weights between features depress, distal weights remain close to the initial value. **c** The final synaptic weight matrix corresponding to panel **b**. **d** A second network consists of two associative memories, “chocolate” and “coffee”. Each association is composed of four features. The associations share two features (“Colour” and “Like”), the connectivity is all-to-all and all weights are initialised at the lower bound. **e**–**g** During the first 100 s, the “chocolate” association is activated ten times more frequently than the “coffee” association. Proximal and distal synaptic weights between neurons of the “chocolate” association are strengthened **e**, **f**. From 100 to 200 s, we reverse the activation probability: the “coffee” association is presented ten times more frequently. The synaptic weights between neurons of this association are now maintained or strengthened **e**, **g**. The proximal synapses towards features that are exclusively part of the “chocolate” association are now weakened, while the corresponding distal synapses remain more stable **f**. During the final 100 s, the same protocol as in the initial 100 s is followed, activating mostly “chocolate” association. The proximal synapses towards features that are exclusively part of the “coffee” association are now weakened, while the corresponding distal synapses remain more stable **g**. **h**, **i** Weight matrix after 200 s (**h** and asterisk in **e**–**g**) and 300 s (**i** and triangle in **e**–**g**) of the simulation. Notice the difference in the proximal weights (left panels) from features “Like” and “Colour” to features “Sweet”,”Food”,”Hot” and “Drink”. The distal weights (right panels) remain similar

To investigate the network effects of dLTP, we implemented a reduced neuron model. In this model, each dendrite consists of only two compartments, one mimicking the proximal part of basal dendrites and the other mimicking distal regions (top in Fig. 6a and see Methods section and Supplementary Fig. 5).

We studied how ongoing activity affects memory retention in networks. In the first network, we implemented four groups of neurons, which we take to represent four features that constitute one association. For example, with “coffee” one could associate the features “drink” ; “colour brown”; “hot”; and “something you like” (bottom in Fig. 6a). The network neurons are all-to-all connected and the connections are randomly distributed across distal and proximal compartments. Importantly, the distal connections coming from neurons of the same feature are always clustered on the same distal compartment post-synaptically. We simulated ongoing activity by randomly choosing a feature and activating it. Carrying on the previous example, we can imagine that we encounter a brown colour, a drink, something hot and the something you like at occasions other than when thinking about coffee. Since the neurons from these different features are never activated together, proximal weights between different features are weakened. However, the active features always stimulate distally projecting clustered synapses, and NMDA spikes will be evoked more easily. As a result, we find that the distal weights between neurons of different features do not depress substantially compared to the proximal weights (Fig. 6b, c).

As in the previous simulation, the ongoing activity might be caused by the activation of different associative memories sharing groups of neurons. We therefore explored how the learning and re-learning of such associations affect each other. We divided a network into two associative memories, for example one “chocolate” and the other “coffee”. Each consists of 4 groups of neurons, representing different features of the association. Both chocolate and coffee share colour and “something you like” features while having two unshared features each, resulting in a total of six features (Fig. 6d).

We hypothesise that dLTP, which allows a neuron to disconnect its output from its learning, enables the retention of previously learned connections in situations where STDP would not. Half of the connections are proximal and half distal. Furthermore, distal connections arriving from neurons that are part of the same feature are clustered onto the same distal compartment of the postsynaptic neurons. In a first phase of the simulation, the “chocolate” association has a 10-fold higher probability of being activated then the “coffee” association. In this phase, both distal and proximal connections between neurons activated by “chocolate” are strengthened (Fig. 6e, f left side). In the second phase, we reverse the probabilities and “coffee” is now ten times more likely to be activated than “chocolate”. Since these memories share half of their neurons, learned connections can be erased. More specifically, when only presynaptic activity is present without postsynaptic activity, synaptic depression is induced in our model (Methods section); hence we expect the connections from the shared neurons to the unshared neurons of the first association to be erased. However, the distal connections allow NMDA spikes to be evoked post-synaptically, so the connections are not depressed (Fig. 6f, middle). This leads to a partial retention of the learned associations, where the distal connections protect previously strengthened synapses from being completely erased. In the final phase, we reversed the probabilities again, observing a similar strong depression of proximal weights from the shared neurons to the unshared neurons of the coffee association, while the distal weights remain close to their values at the start of the phase (Fig. 6g, right).

Our simulations suggest that dLTP allows a subset of strengthened weights to be maintained for a longer time compared to STDP. Due to this mechanism, a trace of a previously learned memory can remain present even when the memory has not been activated for a long time. dLTP protects the weights from being weakened by ongoing activity, while synapses unable to evoke dLTP are depressed. The strength of the latter type of synapses therefore depends on their more recent activation history, favouring associations which are ’fresher’ in the memory.

## Discussion

We investigated how dendrites allow for various plasticity mechanisms within a neuron, and how these depend on the location of the synapse along the dendrite. In our computational model, STDP is present across the whole-basal dendritic tree, although potentiation is attenuated distally due to the attenuation of bAPs^44, 53^. dLTP occurs in about half of the basal dendritic tree and usually requires fewer inputs compared to STDP. This mechanism allows for potentiation even when inputs are unable to evoke action potentials and requires that functionally related synapses are found in nearby locations. It is unclear to what extent such synaptic clusters are present^15, 54–57^. The typical length along which calcium signals spread and dLTP can occur is around 10–20 microns^35, 58, 59^, which suggests that synapses can communicate directly on this scale without the need for action potentials. This form of potentiation would need as few as two to four synapses along a 20 micron stretch of dendritic length^35^. Considering a spine density of about 1–1.5 spines per micron^60^, such clusters need not be apparent and neighbouring spines can easily form synapses from distinct signals. Moreover, this local cooperative potentiation of nearby synapses could be restricted to early postnatal development^61^. In order to arrange synapses with the necessary spatial proximity, structural plasticity might play an additional important role^54^, as shown by theoretical models^62, 63^.

Homoeostatic mechanisms could also provide a strengthening of inputs which are unable to evoke action potentials, but in an unspecific manner^64^. This is in contrast with the highly specific potentiation of locally cooperating spines. While the generation of NMDA spikes is required for this type of potentiation with the current model parameters, a recent study in the hippocampus revealed dLTP occurring without dendritic spikes^35^. To account for this type of potentiation, we simply would need to refine our plasticity rule. We remark that the resulting power of dLTP relative to STDP would be further increased, as less synaptic input would be required by the former. This plasticity mechanism could additionally be gated by brain-derived neurotrophic factor (BDNF), as observed in Gordon et al.^30^. In our model, synaptic potentiation and depression are not quite balanced at distal locations. It would be interesting to investigate how LTP depends on the duration of an NMDA spike and how it compares to STDP, allowing us to further refine our plasticity model. Furthermore, the effects of synaptic location on STDP are more prominent at low pairing frequencies in experiments compared to our model^19, 21^. However, experimentally a spike might be induced by a strong extracellular shock, which could emphasise the difference between proximal and distal locations even at low rates due to the voltage-dependence of plasticity.

Our results show substantially different connectivities arising from clustered synapses compared to unclustered synaptic arrangements. If a substantial amount of clustering is observed, we predict that distally clustered synapses can strengthen even at low rates. This would imply an over-representation of bidirectional connections for neurons connected with distal clusters, while proximally connected neurons would express more unidirectional connections, consistent with Markram et al.^52^. In the absence of clusters, we predict that distal synapses need higher rates than proximal synapses to be strengthened. A similar conclusion is reached in modelling work by Kumar et al.^65^, where it was shown that individual synapses are tuned to an optimal input frequency, which is higher for distal synapses. While distance-dependent synapse densities and synapse strengths have been ovserved in the hippocampus^66, 67^, it would be interesting to see if future experimental data shows a dependency of input rate and connectivity on the synaptic distance from the soma.

We further showed how the plateau depolarisation in the soma, caused by dendritic NMDA spikes, enables other weak inputs to reach the spiking threshold. In this way, the more distally located inputs can determine the direction of plasticity at other synapses, by having the dendritic spike as a teacher signal. Similar mechanisms have been reported in hippocampus^32, 68, 69^ and explored in theoretical models^70^. Moreover, it has been shown that the modulation of gain curves between different regions on a single branch is asymmetric and location dependent^71^, revealing a complex interplay between various inputs.

Finally, we showed in networks of reduced neurons^70–78^ that dLTP can prolong the retention of associative memories. Note that when we speak about memory retention in this article, we mean the protection against being overwritten by other activity, not synaptic consolidation^79^. In our model unclustered synapses weaken faster than clustered synapses, and although we assumed functional clustering of distal inputs from the start, the formation of such clusters might also require a longer time than the formation of unclustered synapses. Similar mixtures of fast and slow learning rates have been discussed in the context of memory retention and the plasticity–stability dilemma^80, 81^. Our results can be regarded as a new implementation of such a combination of learning rates. A distal cluster could play a similar role to synaptic consolidation, and could therefore help in the trade-off between plasticity and stability.

In this study, there was a variety of plasticity mechanisms we did not consider, specifically, intrinsic and homoeostatic plasticity^82–85^, structural plasticity^86^, heterosynaptic plasticity^87, 88^ and branch strength plasticity^72, 89, 90^. Moreover, we only considered basal dendrites, and different learning rules might be present in apical tuft dendrites^33, 36, 91, 92^. Furthermore, we neglected inhibitory synapses, which are known to target specific regions of the dendritic tree^93, 94^, affect the neuronal output in a location-dependent way^73, 95^, and influence the back-propagating action potential and hence synaptic plasticity^96^.

In conclusion, our simulations confirm that in the distal regions on basal dendrites, a small number of synapses can cooperate to evoke NMDA spikes and trigger LTP. This type of potentiation is independent of action potentials and therefore decouples learning from neuronal output. We showed that the connectivity between pairs of neurons is highly influenced by their synaptic arrangement, and made experimentally testable predictions on the location dependence of unidirectional and bidirectional connections. We also investigated how NMDA spikes can gate plasticity at other synapses, and how longer retention of learned associations arises in networks of neurons with active dendrites, while still allowing the same neurons to form new associations.

## Methods

For all simulations of the biophysical neurons, we used the Brian2 neuron simulator^46^ in Python. The network simulations were developed in Python.

### Neuron morphology and passive properties

We used a morphological reconstruction of a Layer 5 and a Layer 2/3 pyramidal neuron of mouse visual cortex, reconstructed by Acker et al.^44^ and Branco et al.^45^, respectively. The HOC files were converted into an SWC file using the free software NLMorphologyConverter (http://neuronland.org/NLMorphologyConverter/NLMorphologyConverter.html). For this purpose, the original soma of the Layer 2/3 model (21 compartments) was replaced by a single-compartment spherical soma with approximately the same volume (radius of the sphere = 8 μm). The total number of compartments in the Layer 5 and Layer 2/3 model were 1181 and 488 respectively. The passive parameters for the neuron can be found in Table 1.

**Table 1:** Passive parameters

Time step	d*t*	25 μs
Leak conductance	*g*_L_	0.04 mS cm^−2^
Leak potential	*E*_L_	−60 mV
Membrane capacitance	*C*_m_	1 μF cm^−2^
Axial resistance	*R*_a_	90 Ω cm

To account for spines, we increased the leak conductance and membrane capacitance by factor of 1.5 for distances larger than 50 microns from the soma, as in Acker et al.^44^. The value of *E*_*L*_ was chosen to ensure a resting potential of the neuron of −69 mV in the Layer 5 and −72 mV in the Layer 2/3 neuron.

### Ion channels

The active conductances in our model were adapted from Acker et al.^44^, a study in which a fit was made to match experimental data on action potential back propagation in basal dendrites of layer 5 pyramidal cells. The most prominent differences between layer 5 and layer 2/3 pyramidal neurons are found in the apical dendrites: the Ca^2+^-spike initiation zone and Ih current are almost completely absent in layer 2/3 neurons^97–99^. These results indicate that the apical tuft dendrites of layer 2/3 neurons might be more similar to the basal dendrites. Nevertheless, we chose to distribute synapses solely across the basal dendritic tree in all our simulations.

We further modified the distribution of the channels in the soma and axon from Acker et al.^44^ in order to have a spike initiation in the initial part of the axon instead of the soma. All equations governing the channel dynamics were identical as the equations in Acker et al.^44^, but translated from the HOC files into python with Brian2. As in Acker et al.^44^, most temperature dependencies are removed from the ion conductances, except for the temperature dependence of the high-threshold calcium channel. The conductance of the latter should therefore be multiplied by a factor of 2.11 for a fair comparison with other conductances, since we used a temperature of 32 degrees Celsius. We point out that in most models with the NEURON simulator, the conductances are temperature dependent, which one should take into account when comparing with the current work. Full details on the ion channel dynamics and distributions can be found in Acker et al.^44^, a summary of the ion channel distributions in our model can be found in Table 2. For simplicity, we use the following abbreviations:

- Voltage-gated Na^+^ channels : Na_v_
- Delayed-rectifier K^+^ channels: K_v_
- A-type K^+^ channel: Ka_v_
- High-threshold Ca^2+^ channels: Cah_v_
- Low-threshold Ca^2+^ channels: Cal_v_

**Table 2:** Ion channels

*Soma*	
Na_v_	200 pS μm^−2^
K_v_	400 pS μm^−2^
Ka_v_ (proximal type)	300 pS μm^−2^
Cah_v_	4 pS μm^−2^
Cal_v_	1 pS μm^−2^
*Basal dendrites (d = distance to the soma in microns)*	
Na_v_	(150–0.5×*d*×μm^−1^) pS μm^−2^
K_v_	40 pS μm^−2^
Ka_v_	(150 + 0.7×*d*×μm^−1^) pS μm^−2^
Cah_v_	0.4 pS μm^−2^
Cal_v_	0.1 pS μm^−2^
*Apical dendrites*	
Na_v_	250 pS μm^−2^
K_v_	40 pS μm^−2^
Ka_v_	300 pS μm^−2^
Cah_v_	0.4 pS μm^−2^
Cal_v_	0.1 pS μm^−2^

The Ka_v_ channel density in all dendrites is further divided into proximal and distal A-type potassium channels (full details can be found in Acker et al.^44^).

In order to mimic spike initiation in the axon, the initial two segments of the axon have a K_v_ density of 40 and 100 pS μm^−2^, respectively, and a Na_v_ density of 32,000 and 28,000 pS μm^−2^, respectively. The rest of the axon has a constant K_v_ density of 500 pS μm^−2^ and Na_v_ density of 20,000 pS μm^−2^. There are no calcium conductances in the axon, except in the first two compartments where they have the same values as in the soma. In addition, a low-threshold activated, slowly inactivating potassium current is added in the first four compartments of the axon, and is zero otherwise. As this study focuses on plasticity in dendrites, the axonal ion distributions were chosen merely to allow for a spike generation and not for biological plausibility.

### Synapses

Synapses consist of both AMPA and NMDA channels. Only the AMPA component was plastic, and its maximal conductance was chosen equal to the NMDA conductance. This ratio is within the experimentally observed range^51^. We refer to the Supplementary Information for simulations with different AMPA to NMDA ratios (Supplementary Fig. 2) and for simulations where both AMPA and NMDA components are plastic (Supplementary Figs 6–10).

An activated synapse will result in an instantaneous rise of both AMPA and NMDA conductances by an amount of *g*(*t*) = *w*(*t*) · *g*_max_, where *w*(*t*) is the synaptic weight and *g*_max_ is the maximal conductance for either AMPA or NMDA. This is followed by an exponential decay with the respective time-constants for AMPA and NMDA:

$$\frac{{{\rm{d}}g(t)}}{{{\rm{d}}t}} = - g(t){\rm{/}}{\tau _{{\rm{AMPA/NMDA}}}} + w(t) \cdot {g_{{\rm{max}}}} \cdot \delta \left( {t - {t_f}} \right)$$

*δ*(*x*) is the Dirac delta function, *t*_*f*_ is the presynaptic firing time and *τ*_AMPA/NMDA_ is the synaptic time constant of AMPA or NMDA receptors. The current flowing through AMPA receptors is modelled by

$${I_{{\rm{AMPA}}}}(t) = g(t) \cdot \left( {u(t) - {E_{{\rm{AMPA}}}}} \right)$$

while the NMDA current is given by

$${I_{{\rm{NMDA}}}}(t) = g(t) \cdot B(u(t)) \cdot \left( {u(t) - {E_{{\rm{NMDA}}}}} \right)$$

*B*(*v*) describes the voltage-dependence of the magnesium block in NMDA channels^100^,

$$B(v) = \frac{1}{{1 + \frac{{{\rm{exp}}\left( { - 0.062 \cdot v} \right)}}{{3.57}}}}$$

All parameters can be found in Table 3.

**Table 3:** Synapses and plasticity

AMPA time-constant	*τ*_AMPA_	2 ms
AMPA reversal potential	*E*_AMPA_	0 mV
NMDA time-constant	*τ*_NMDA_	50 ms
NMDA reversal potential	*E*_NMDA_	0 mV
maximal AMPA conductance	*g*_AMPA_	1.5 nS
NMDA conductance	*g*_NMDA_	1.5 nS
Low-pass filter time constant 1	*τ*_1_	5 ms
Low-pass filter time constant 2	*τ*_−_	15 ms
Low-pass filter time constant 3	*τ*_+_	45 ms
Depolarisation threshold for plasticity	*θ*_−_	−72 mV (L2/3), −69 mV (L5)
Depolarisation threshold for potentiation	*θ*_+_	−15 mV
Spike trace reset value	*x*_reset_	5
Spike trace time constant	*τ*_*x*_	20 ms
Depression amplitude	*A*_LTD_	4e−4 mV^−1^
Potentiation amplitude	*A*_LTP_	14e−4 mV^−2^

### Plasticity

The plasticity rule used is based on the voltage-based STDP rule from Clopath et al.^27^ without homoeostasis, where the synaptic weights *w*_*i*_ follow

$$\frac{{{\rm{d}}{w_i}(t)}}{{{\rm{d}}t}} = - {A_{{\rm{LTD}}}}{X_i}{\left( {{{\bar u}_ - }(t) - {\theta _ - }} \right)_ + } + {A_{{\rm{LTP}}}}{\bar x}_{i}(t){\left( {u(t) - {\theta _ + }} \right)_ + }{\left( {{{\bar u}_ + }(t) - {\theta _ - }} \right)_ + }$$

with

$$\begin{array}{l}\\ {\tau _x}\frac{{{\rm{d}}{{\bar x}_i}(t)}}{{{\rm{d}}t}} = - {{\bar x}_i}(t) + {X_i}(t)\\ \\ {\tau _ - }\frac{{{\rm{d}}{{\bar u}_ - }(t)}}{{{\rm{d}}t}} = - {{\bar u}_ - }(t) + {{\bar u}_1}(t)\\ \\ {\tau _ + }\frac{{{\rm{d}}{{\bar u}_ + }(t)}}{{{\rm{d}}t}} = - {{\bar u}_ + }(t) + {{\bar u}_1}(t)\\ \\ {\tau _1}\frac{{{{\bar u}_1}(t)}}{{{\rm{d}}t}} = - {{\bar u}_1}(t) + u(t)\\ \end{array}$$

All parameters were in the model were chosen manually in order to qualitatively reproduce the plasticity experiments in Sjöström et al.^23^ at both proximal and distal locations (Fig. 2). We were especially interested in the differences between proximal and distal locations and therefore we aimed for a qualitative agreement with the experimental data. The most important difference with the original model^27^ is that the voltage *u*(*t*) used for our implementation is the local voltage at the dendritic compartment, and not the somatic voltage. The voltage *u*(*t*) in each compartment is generated by the Brian2 simulator.

Another difference with the original model of Clopath et al.^27^ is the extra low-pass filter ${\bar u_1}$, implemented to introduce a small delay in the low-pass filters ${\bar u_ + }$ and ${\bar u_ - }$. This was achieved in the original model by filtering *u*(*t* − $\epsilon$).

We refer the reader to ref. ^27^ for full details and give a short summary here: this rule has a depression term and a potentiation term. The term responsible for depression incorporates a presynaptic spike train *X*_*i*_ and the low-pass filtered postsynaptic depolarisation ${\left( {{{\bar u}_ - } - {\theta _ - }} \right)_ + }$. The (·)_+_ stands for rectification, i.e., the term is only non-zero if the number within the brackets is positive. ${\bar u_ - }$ is a low-pass filtered version of the membrane potential with time constant *τ*_−_, and *θ*_−_ is a threshold for synaptic plasticity. To summarise, this term states that a presynaptic spike will lead to depression if the postsynaptic membrane is depolarised, for example because the postsynaptic neuron fired a spike in the recent past. An isolated post–pre pairing will therefore lead to depression. The term responsible for potentiation includes a low-pass filtered presynaptic spike train ${\bar x}_{i}$, a low-pass filtered postsynaptic depolarisation ${\left( {{{\bar u}_ + } - {\theta _ - }} \right)_ + }$ and an instantaneous depolarisation above a potentiation threshold (*u* − *θ*_+_)_+_. The time constant for the low-pass filtered version of the membrane potential ${\bar u_ + }$ is given by *τ*_+_. The potentiation threshold *θ*_+_ is high enough to be selective for back-propagating action potentials or NMDA spikes. Potentiation can therefore occur when a postsynaptic spike happens after a presynaptic neuron fired and while the postsynaptic membrane is depolarised, for example because the postsynaptic neuron fired another spike in the recent past. Potentiation is therefore possible when there are triplets of spikes (pre–post–post or post–pre–post), which can occur as isolated triplets or during a sustained high rate firing. Second, potentiation can occur when an NMDA spike (also called dendritic plateau potential) is generated. These plateau potentials provide a long and sufficiently high depolarisation, leading to potentiation without generating postsynaptic action potentials. The above equations ensure all-to-all plasticity. The parameters were chosen to qualitatively reproduce the frequency dependence of plasticity^23^, and are listed in Table 3.

In all simulations, the minimum and maximum weight for the synapses were 0.01 and 1 respectively, unless stated otherwise. The choice of *θ*_+_, the threshold for potentiation, was made by comparing data from Sjöström et al.^20^ with data from Acker et al.^44^. In the former, it is shown how the attenuation of the bAP results in a transition from potentiation to depression at a distance of about 200 μm, while in the latter it is shown that the bAP amplitude at this distance is roughly 60 mV above the resting potential (−69 or −72 mV in for the layer 5 and layer 2/3 neurons, respectively).

We want to point out that all parameters and equations used in our models are the result of fitting procedures or aimed to qualitatively reproduce experimental observations. In doing so, simplifications are necessarily introduced and therefore we do not claim that any value represents a ’real’ biologically correct value. Instead, the parameters should be interpreted as a set of abstractions that enable a qualitative reproduction of biological phenomena relevant for the scope of this article.

### Reduced multicompartmental model

The reduced compartmental model was implemented in Python. For this purpose, a somatic compartment was coupled to two compartments per dendrite: one representing the proximal section and one the distal section.

The compartments are coupled through their voltage difference and parameters representing axial resistance (details below). The parameters were chosen to reproduce the L5 detailed model phenomenologically, and might not be the only set of parameters satisfying this behaviour. A comparison between both models can be found in Supplementary Fig. 5.

Each compartment is modelled using an integrate-and-fire model, based on Clopath et al.^27^ and where the membrane potential *u*(*t*) of the compartment is calculated as

$$C\frac{{{\rm{d}}u(t)}}{{{\rm{d}}t}} = {I_L}(t) + {I_{{\rm{exp}}}}(t) + {I_{{\rm{axial}}}}(t) + {I_{{\rm{ext}}}}(t) + {I_{{\rm{noise}}}}(t)$$

Where the leak current *I*_*L*_, the exponential current *I*_exp_, axial currents *I*_axial_ and a noise current *I*_noise_ are given by

$$\begin{array}{l}\\ {I_L}(t) = - {g_L}\left( {u(t) - {E_L}} \right)\\ \\ {I_{{\rm{exp}}}}(t) = {g_L}{\Delta _T}\,{\rm{exp}}\left( {\frac{{u(t) - {V_T}(t)}}{{{\Delta _T}}}} \right)\\ \\ {I_{{\rm{axial}}}}(t) = - \mathop {\sum}\limits_i {g_i} \cdot \left( {u(t) - {u_i}(t)} \right)\\ \\ {I_{{\rm{noise}}}}(t) = \sqrt {2{\tau _{{\rm{noise}}}}{\rm{/ms}}} \cdot s(t) \cdot {\sigma _{{\rm{noise}}}} + {\mu _{{\rm{noise}}}}\\ \end{array}$$

where C is the membrane capacitance, *g*_*L*_ is the leak conductance and *E*_*L*_ is the resting potential. *I*_ext_ is an external current, *I*_inhib_ is an inhibitory current described below. The exponential current models the fast sodium activation in the soma and is not present in the dendritic compartments. Δ_*T*_ is the slope factor for the exponential term describing the sodium current activation, *V*_*T*_ is an adaptive threshold potential which starts at *V*_*T*max_ after a spike and decays to *V*_*T*rest_:

$${\tau _{{V_T}}}\frac{{{\rm{d}}{V_T}(t)}}{{{\rm{d}}t}} = - \left( {{V_T}(t) - {V_{{T_{{\rm{rest}}}}}}} \right)$$

The current entering from or leaving to neighbouring compartments is given by *I*_axial_. The voltage difference between the compartment and each neighbour is multiplied by an appropriate conductance, chosen to reproduce the interaction between soma,proximal and distal compartments of the detailed model. The values for the conductances between soma-proximal and proximal-distal compartments are given below. In order to mimic the difference in efficiency between forward and backward propagation of currents, the conductances are different for both directions:

$$\begin{array}{*{20}{l}}\\ {{I_{{\rm{axial,soma}}}}}={ - \mathop {\sum}\limits_i {(50\,{\rm{nS}}) \cdot {{\left( {{u_{{\rm{soma}}}} - {u_{{\rm{prox,i}}}}} \right)}_ - }} } \hfill \\ \\ {{I_{{\rm{axial,prox}}}}} =  { - \left( {1250\,{\rm{nS}}} \right) \cdot {{\left( {{u_{{\rm{soma}}}} - {u_{{\rm{prox}}}}} \right)}_ - } - (2500\,{\rm{nS}}) \cdot {{\left( {{u_{{\rm{soma}}}} - {u_{{\rm{prox}}}}} \right)}_ + }} \hfill \\ \\ {}  {} \;\;\;\;\;\;\;\;\;\;\;\;\;\;\;\; { - (225\,{\rm{nS}}) \cdot {{\left( {{u_{{\rm{prox}}}} - {u_{{\rm{dist}}}}} \right)}_ - } - (1500\,{\rm{nS}}) \cdot {{\left( {{u_{{\rm{prox}}}} - {u_{{\rm{dist}}}}} \right)}_ + }} \hfill \\ \\ {{I_{{\rm{axial,dist}}}}} = { - (1500\,{\rm{nS}}) \cdot {{\left( {{u_{{\rm{dist}}}} - {u_{{\rm{prox}}}}} \right)}_ + } - (225\,{\rm{nS}}) \cdot {{\left( {{u_{{\rm{dist}}}} - {u_{{\rm{prox}}}}} \right)}_ - }} \hfill \\ \end{array}$$

Where we dropped the dependence on time for clarity and (·)_+_ is only non-zero if the value within the brackets is positive, while (·)_−_ is only non-zero if the value within the brackets is negative.

The current *I*_noise_ is coloured noise with standard deviation *σ*_noise_ and mean *μ*_noise_. *τ*_noise_ is the time-constant for the low-pass filtering of the noise and *s*(*t*) is the low-pass filtered version of a Gaussian white noise *ξ*(*t*) with zero mean and unit s.d.:

$${\tau _{{\rm{noise}}}}\frac{{{\rm{d}}s(t)}}{{{\rm{d}}t}} = - s(t) + \xi (t)$$

The spike heights at proximal and distal compartments were chosen by taking the mean of spike heights in the compartments of the detailed model shown in Fig. 1a. When the voltage in the somatic compartment reaches the spiking threshold (20 mV), a spike is simulated by holding the somatic voltage at 30 mV during 1 ms. After the spike, the somatic voltage is reset to the reset value *V*_reset_. For every somatic spike, the back propagation into the dendrites is modelled by holding the dendritic potential at 10 mV during 1 ms in the proximal compartments, and at −3 mV during 1 ms in the distal compartments, and with an additional delay compared to the somatic spike of 0.3 ms. All other parameters can be found in Table 4.

**Table 4:** Reduced neuron parameters

Timestep	d*t*	250 μs
Leak conductance	*g*_*L*_	40 nS
Resting potential	*E*_*L*_	−69 mV
Membrane capacitance	*C*_*m*_	281 pF
AMPA time constant	*τ*_AMPA_	2 ms
AMPA reversal potential	*E*_AMPA_	0 mV
NMDA time constant	*τ*_NMDA_	50 ms
NMDA reversal potential	*E*_NMDA_	0 mV
Maximal AMPA conductance	*g*_AMPA_	100 nS
NMDA conductance	*g*_NMDA_	50 nS
Spiking threshold	*θ*	20 mV
Slope factor	Δ_*T*_	2 mV
Adaptive threshold rest value	*V*_*T*rest_	−50.4 mV
Adaptive threshold max value	*V*_*T*max_	−30.4 mV
Adaptive threshold time constant	*V*_*T*tau_	50 ms
Spike width	/	1 ms
Voltage reset after spike	*V*_reset_	−55 mV
Low-pass filter time constant 1	*τ*_−_	35 ms
Low-pass filter time constant 2	*τ*_+_	35 ms
Depolarisation threshold for plasticity	*θ*_−_	−69 mV
Depolarisation threshold for potentiation	*θ*_+_	−15 mV
Spike trace reset value	*x*_reset_	1
Spike trace time constant	*τ*_*x*_	15 ms
Depression amplitude	*A*_LTD_	5e−4 mV^−1^
Potentiation amplitude	*A*_LTP_	15e−4 mV^−2^

Similar to the detailed model, synapses consist of both AMPA and NMDA channels, and only the AMPA component is plastic. An activated synapse will result in an instantaneous rise of the AMPA and NMDA conductances by an amount of *g*(*t*) = *w*(*t*) · *g*_max_, where w is the synaptic weight and *g*_max_ is the maximal conductance for either AMPA or NMDA. This is followed by an exponential decay with the respective time-constants for AMPA and NMDA. All parameters are in listed Table 4. We mimic the increased input resistance at distal synapses by multiplying the AMPA and NMDA currents at these compartments by a factor of 2.5.

The plasticity rule in the reduced model is exactly as in the detailed model, with the only difference that ${\bar u_ - }$ and ${\bar u_ + }$ are defined in the following way:

$$\begin{array}{l}\\ {\tau _ - }\frac{{{\rm{d}}{{\bar u}_ - }(t)}}{{{\rm{d}}t}} = - {{\bar u}_ - }(t) + u(t - \epsilon )\\ \\ {\tau _ + }\frac{{{\rm{d}}{{\bar u}_ + }(t)}}{{{\rm{d}}t}} = - {{\bar u}_ + }(t) + u(t - \epsilon )\\ \end{array}$$

Hence, a small delay is introduced differently in the models: in the reduced model, $\epsilon$ is equal to the width of an action potential, 1 ms. In the full model, a delay is introduced by an extra low-pass filter ${\bar u_1}$ which is absent in the reduced model. To ensure that the observed effects are not due to a disproportionately strong dLTP, but rather due to the decoupling of the learning from the neuronal output by dLTP, we multiplied the amplitude of dLTP by a factor of 0.15 and achieved a better balance between distal depression and potentiation. This was achieved by storing the local voltage value of each compartment during 1.3 ms. We then compared whether the actual value of the local voltage and the saved value 1.3 ms earlier were both above the potentiation threshold *θ*_+_. Since the spike width was 1 ms in our model, this situation could only be caused by an NMDA spike and in that case the potentiation amplitude was reduced. In principle, one could imagine firing rates above 500 Hz satisfying the same condition, however the neurons in our network fired at lower rates. All parameters can be found in Table 4.

To allow higher rates without leading to unbounded activity in our recurrent networks, we approximated an inhibitory current at the soma in the following way. For each neuron, the spikes of all its presynaptic neighbours are filtered with a time constant *τ*_inhib_ of 30 ms:

$$\frac{{{\rm{d}}{E_{{\rm{in}}}}(t)}}{{{\rm{d}}t}} = - {E_{{\rm{in}}}}(t){\rm{/}}{\tau _{{\rm{inhib}}}} + \mathop {\sum}\limits_i \delta \left( {t - {{t}_{i}^{f}}} \right)$$

Where $t_i^f$ are the firing times of presynaptic neurons. A short rise time for the inhibition is achieved by filtering *E*_in_ with a time constant *τ*_rise_ of 2 ms:

$$\frac{{{\rm{d}}{g_{{\rm{in}}}}(t)}}{{{\rm{d}}t}} = \left( { - {g_{{\rm{in}}}}(t) + {E_{{\rm{in}}}(t)}} \right){\rm{/}}{\tau _{{\rm{rise}}}}$$

This filtered trace of incoming excitatory activity *g*_in_ is transformed as follows:

1 $${I_{{\rm{inhib}}}}(t) = - {A_{{\rm{inhib}}}} \cdot {g_{{\rm{in}}}}(t) \cdot \left( {{u_{{\rm{soma}}}}(t) - {E_{{\rm{GABA}}}}} \right)$$

where *I*_inhib_ is the inhibitory current arriving at the somatic compartment, *A*_inhib_ is a scaling constant with value 125 pS for Fig. 6b, c and 100 pS for Fig. 6e–i, *u*_soma_ is the somatic voltage and *E*_GABA_ = −75 mV is the reversal potential for GABA.

### Analysis of existing data from Markram et al

We reanalysed the data reported in Table 3 in Markram et al.^52^. We only used basal and oblique dendrite data, since the plasticity at distal tuft dendrites could depend on calcium spikes^91^.

The values for the distance from the soma for unidirectionally connected neurons are

14.7 μm, 74.4 μm (7×), 75.8 μm (14×), 117.7 μm, 91.3 μm (4×), 134 μm (6×), 162.6 μm

Resulting in an average of (± one s.d.):

89.6 μm±28.6 μm

The values for the distance from the soma for bidirectionally connected neurons are

73.3 μm, 75.6 μm (10×), 86.9 μm (23×), 97 μm (10×), 184.6 μm (3×), 157 μm (10×), 141.8μm (5×)

Resulting in an average of (± one s.d.):

106.9 μm±33.7 μm

### Figure 1

*Figure* *1a*: 17 distal and 17 proximal compartments in the neuron model are identified. The distal compartments are further split into two groups: one group consists of distal compartments eliciting NMDA spikes when activating synaptic input as described for Fig. 1b below, and the other group consists of compartments where no NMDA spike is elicited with such input. We considered an NMDA spike to be evoked if the local depolarisation crossed a threshold at −15 mV. Using this criterion, 12 out of the 17 distal compartments where classified in the first group and marked with red in the figure. All 17 proximal compartments are shown in blue.

*Figure* *1b*: to all compartments shown in Fig. 1a, a synapse at the weight *w*_max_ = 1 was connected. For each compartment, the following simulation was performed: the connected synapse was activated 10 times with an interspike interval of 1 ms while storing the membrane potential at the soma. This protocol was repeated for interspike intervals of 4 ms and 8 ms. Figure 1b shows the mean (solid line) and standard deviation (shaded region) of the somatic potential calculated for all proximal compartments (blue) and distal compartments (red). The AMPA and NMDA components were not plastic and both conductances were 2 nS.

*Figure* *1c, d*: For each compartment of the basal dendrites in our morphological neuron, a number of synapses with weights fixed at *w* = 1.5 were activated with interspike intervals of 1 ms and the local membrane voltage was recorded during the subsequent 50 ms. The simulation was repeated with an increased number of synapses until a threshold at −15 mV was crossed. By comparing the local voltage with the somatic voltage, it was determined whether this threshold was crossed due to an NMDA spike or a somatic spike. The number of synapses needed to reach this threshold was stored.

### Figure 2

*Figure* *2b*: At the same compartments as shown in Fig. 1a, a synapse is activated reproducing the experimental protocol in^23^. Pairs of pre–post or post–pre spikes separated by 10 ms are repeated a further five times at different frequencies of 5 Hz up to 50 Hz with steps of 5 Hz, and for 1 Hz. The weight change is multiplied by a factor of 15 to mimic the experimental protocol in Sjöström et al.^23^. The initial synaptic weights are 0.5 and the weight change after the six pairings are plotted in function of the frequency. The solid lines represent the means of the simulations in all proximal compartments (blue) or distal compartments (red). The shaded areas represent one s.d.

*Figure* *2c*: The same pairing protocol as in Fig. 2b at 20 Hz was repeated for different interspike intervals (1, 2.5, 5, 7.5, 10, 12.5, 15.17.5 and 20 ms).

*Figure* *2e*: The synapse in one distal compartment is activated 10 times with an interspike interval of 0.1 ms and weight *w* = 1.

*Figure* *2f*: The compartments shown in Fig. 1a are connected with 10 synapses each, all with an initial weight of 0.5. The 10 synapses are activated using a Poisson process with the same average rate, lasting 200 ms. This protocol is repeated with various rates between 1 Hz and from 10 to 70 Hz with steps of 10 Hz. The solid lines represent the means of the simulations in all proximal compartments (blue) or distal compartments (red). The shaded areas represent one s.d.

### Figure 3

We implemented two layer 5 biophysical neurons (using the same morphology) for all simulations. All synaptic weights are initiated at 0.5, the maximal AMPA conductance and the NMDA conductance were equal to 1 nS. A potentiated synapse was defined as a synapse that had a larger weight value at the end of the simulation compared to the initial value. Bidirectionally connected neurons were defined as neurons where the mean of synaptic weights to both neurons was larger at the end of the simulation compared to the start. Unidirectionally connected neurons were defined as neurons where only the mean of the synaptic weights to one neuron was larger at the end compared to the start, while the mean of the weights to the other neuron was smaller at the end compared to the start. Unconnected neurons were defined as neurons where the mean to both neurons was smaller compared to the initial value. We realise that looking at the mean value of the weights to discriminate between unidirectional and bidirectional is not completely accurate (one can imagine strengthening of some weights and weakening of others in some situations), but it gives us an adequate way of comparing the synaptic weight changes for the different protocols. Action potentials in the neurons were evoked by injecting a brief current into the soma, with an instantaneous rise of 15 nA and an exponential decay back to zero with a time constant of 0.1 ms.

*Figure* *3a–d*: Each neuron made 15 synaptic connections onto its partner. The synaptic locations were drawn randomly across the whole-basal tree. In both neurons, spikes were induced following a Poisson point process with a fixed average rate, and the neurons were simulated for 5 s. We then repeated the simulation for all combinations of rates between 0 and 20 Hz, with 2 Hz steps, and stored all the final synaptic weights for every case. We then repeated the whole protocol 20 times with different randomly drawn synaptic locations, which we also stored.

(b) For each of the 15 simulated pairs of neurons, we calculated the mean synaptic distance from the soma and the amount of potentiated synapses present across all combinations of firing rates.

(c, d) Two examples of simulation outcomes for different randomly chosen synaptic locations. See above for our definition of unidirectional and bidirectional connections.

*Figure* *3e–h*: Each neuron made 10 synaptic connections on its partner. The synaptic locations were drawn randomly across either the proximal or distal compartments as shown in Fig. 1a. In both neurons, spikes were induced following a Poisson point process with a fixed average rate, and we simulated the neurons for 5 s. We then repeated the simulation for all combinations of rates between 0 and 20 Hz, with 2 Hz steps, and stored all the final synaptic weights for every case. We then repeated the whole protocol five times with different randomly drawn synaptic locations, which we also stored.

(f) For each of the five simulated pairs of neurons, we counted the number of unidirectional and bidirectional connections (See above for our definition of unidirectional and bidirectional connections.).

(g, h) Two examples of simulation outcomes for different random synaptic locations across proximal compartments (g) or distal compartments (h). See above for our definition of unidirectional and bidirectional connections.

*Figure* *3i–l*: Exactly the same as e–h, but with the following difference: only one synaptic locations was drawn randomly from either the proximal or distal compartments shown in Fig. 1a, and all 10 synapses were clustered onto that compartment.

*Figure* *3m–n*: All basal compartments are classified into groups according to the distance from the soma, each group ranging across 25 microns, except for the final group which comprised of 33 microns. For each distance range, two neurons are interconnected by 10 synapses each. The synapses are either randomly distributed over compartments in the respective range, or clustered onto one compartment. In both neurons, spikes were induced following a Poisson point process with a fixed average rate, and we simulated the neurons for 5 s. We then repeated the simulation for all combinations of rates between 0 and 20 Hz, with 2 Hz steps, and in each case, we stored all the final synaptic weights. We repeated the whole protocol six times (three for the clustered and three for the distributed case) with different randomly drawn synaptic locations. We counted the amount of unidirectional or bidirectional connections (see above for our definition of unidirectional and bidirectional connections.). To account for the different number of compartments at different distances from the soma, we multiplied the resulting numbers in each distance range by the respective number of compartments.

*Figure* *3o–r*: Each neuron made 10 synaptic connections on its partner. Only one synaptic locations was drawn randomly from either the proximal or distal compartments shown in Fig. 1a, and all 10 synapses were clustered onto that compartment. To simulate temporal order in the activity, we induced spikes only during 10 ms in the first neuron, followed by spikes in the subsequent 10 ms in the second neuron. These 20 ms were then followed by 250 ms without any activity, and the whole 270 ms were repeated 10 times. The induction of spikes in the first and second neuron during the first 10 ms and the second 10 ms, respectively, were achieved by simulating a Poisson process with average rate of 150 Hz. We point out that although this seemingly high firing rate was only sustained for 10 ms (thus on average 1.5 spikes during these 10 ms), and over the whole simulation each neuron had an average rate of 5.5 Hz. The whole protocol described above was repeated five times with different randomly drawn locations.

(p) For each of the five simulated pairs of neurons, we counted the amount of unidirectional or bidirectional connections (see above for our definition of unidirectional and bidirectional connections.). Note that we only repeated this simulation for one activation rate as opposed to the 55 combinations of rates in the previous simulations.

(q, r) The evolution of mean synaptic weights for one example pair of neurons, in the case where the clusters are proximal (q) or distal (r).

### Figure 4

Three input pools consisting of 10 randomly distributed synapses each are initiated at the minimum weight. Five extra synapses are clustered at a distal compartment on a basal dendrite. These synapses are initiated at the maximum weight and will be paired with one of the other input pools. The activation of the pools or the distal cluster comprises of activating the relevant synapses during 250 ms at an average rate of 40 Hz. Subsequent activations of pools were separated by a 200 ms interval. The maximal weight was reduced to 0.5 instead of 1.

During the first half of the simulation (0–45 s), the activations of the first pool are paired with the activation of the distal cluster. During the second half of the simulation (45–90 s), the second pool is paired with the cluster. In order to observe the depression when a pool is not paired with the cluster, the maximum weight is reduced to 0.4 instead of 1. The minimum weight is increased from 0.01 to 0.3, enabling the weakest synapses to still reach the spiking threshold when paired with the cluster. To compensate for the reduced range between the minimum and maximum weight, the amplitudes for depression and potentiation, *A*_*LTD*_ and *A*_*LTP*_, reported earlier are multiplied by a factor of 0.3.

### Figure 5

*Figure* *5a–c*: Two pools of 4 neurons are connected to proximal compartments on basal dendrites (synapses of the same pool are clustered onto the same compartment). The initial weights are at the maximum hard bound 1, and an additional coloured noisy current is injected in the soma:

$$\frac{{{\rm{d}}{I_{{\rm{noise}}}}}}{{{\rm{d}}t}} = \frac{{\left( {{\mu _{{\rm{noise}}}} - {I_{{\rm{noise}}}}} \right)}}{{{\tau _{{\rm{noise}}}}}} + \xi {\sigma _{{\rm{noise}}}}\sqrt {\frac{2}{{{\tau _{{\rm{noise}}}}{\rm{/ms}}}}} $$

with standard deviation *σ*_noise_ = 3.5 pA, mean *μ*_noise_ = 35 pA and *τ*_noise_ = 20 ms is the time-constant for the low-pass filtering of the Gaussian white noise with zero mean and unit standard deviation *ξ*(*t*). We alternate the activation of the two pools, with an activation event comprising of Poisson-distributed spikes at an average rate of 40 Hz at the member synapses during 50 ms. Two subsequent activations are separated by a 150 ms interval. Every 20 activations (i.e., 10 for each pool), both pools are activated simultaneously.

*Figure* *5d–f*: Analogous to Fig. 5a–c, but the two pools are now connected to distal compartments on the basal dendrites. Note that we chose two distal compartments that reliably induced somatic spikes when simultaneously activated. Some other choices failed to do so, but our main result on memory retention of the distally clustered synapses remained valid in all cases.

### Figure 6

For all simulations of Fig. 6, each neuron had 15 dendrites.

*Figure* *6b–d*: We implemented 40 neurons, which we divided into 4 groups of 10 neurons. The neurons are all-to-all connected, with weights initiated at the maximum bound for each simulation, and half of the connections are distal while the other half are proximal. The distal connections coming from neurons of the same feature are always clustered on the same distal compartment post-synaptically. In all neurons, a coloured noisy current was injected in the soma to induce more variability (mean = 150 pA, std = 15 pA). Moreover, each neuron had 50 extra non-plastic synapses that were used as inputs.

(b, c) We activated the input synapses of one of the groups of 10 neurons (the group was selected randomly) by activating the input synapses to the relevant neurons at an average rate of 350 Hz during 10 ms, followed by a 250 ms interval without inputs. We repeated this for 100 s, and randomly chose one of the groups for every activation. The total synaptic strength of the network was stored every second.

*Figure* *6e–j*: We divided a network of 60 neurons into 2 groups of 40 neurons each (’associative memories’), each group sharing 20 neurons. Moreover, the groups were divided into 4 subgroups of 10 neurons (’features’), and two of these subgroups were shared. The neurons were all-to-all connected, with weights initiated at the minimum bound for each simulation, and half of the connections are distal while the other half are proximal. The distal connections coming from neurons of the same feature were always clustered on the same distal compartment post-synaptically. In all neurons, a coloured noisy current was injected in the soma to induce more variability (mean = 150 pA, std = 15 pA). Moreover, each neuron had 50 extra non-plastic synapses that were used as inputs.

An activation event consisted of selecting one associative memory and activating the input synapses to the 40 relevant neurons at an average rate of 350 Hz during 10 ms, followed by a 250 ms interval without input. The selection of the associative memory was done randomly, but the probabilities changed during the simulation: during the first 100 s, the first and second associative memories had 90 and 9% chance of activation respectively, while between 100 and 200 s the probabilities were reversed (9 and 90%, respectively). Finally, between 200 and 300 s the activation probabilities were as in the first phase (90 and 9%, respectively). The total synaptic strength from subgroups 4 to subroups 1 and 6, and from subgroup 3–4 were stored every second. Moreover, the total synaptic weight matrix was stored after 200 and 300 s.

### Data availability

The codes of our models and simulations will be posted on ModelDB (https://senselab.med.yale.edu/modeldb/) after publication.

## Electronic supplementary material

Supplementary Information
