# Supplementary material for: Modeling somatic and dendritic spike mediated plasticity at the single neuron and network level
Source: Nat Commun. 2017 Sep 26;8:706. doi: 10.1038/s41467-017-00740-z (PMC5615054; doi:10.1038/s41467-017-00740-z)
Supplement: Supplementary file 1 — Supplementary Information [file 41467_2017_740_MOESM1_ESM.pdf]

File Name: Supplementary Information
Description: Supplementary Figures, Supplementary Methods and Supplementary Reference

Supplementary Figures

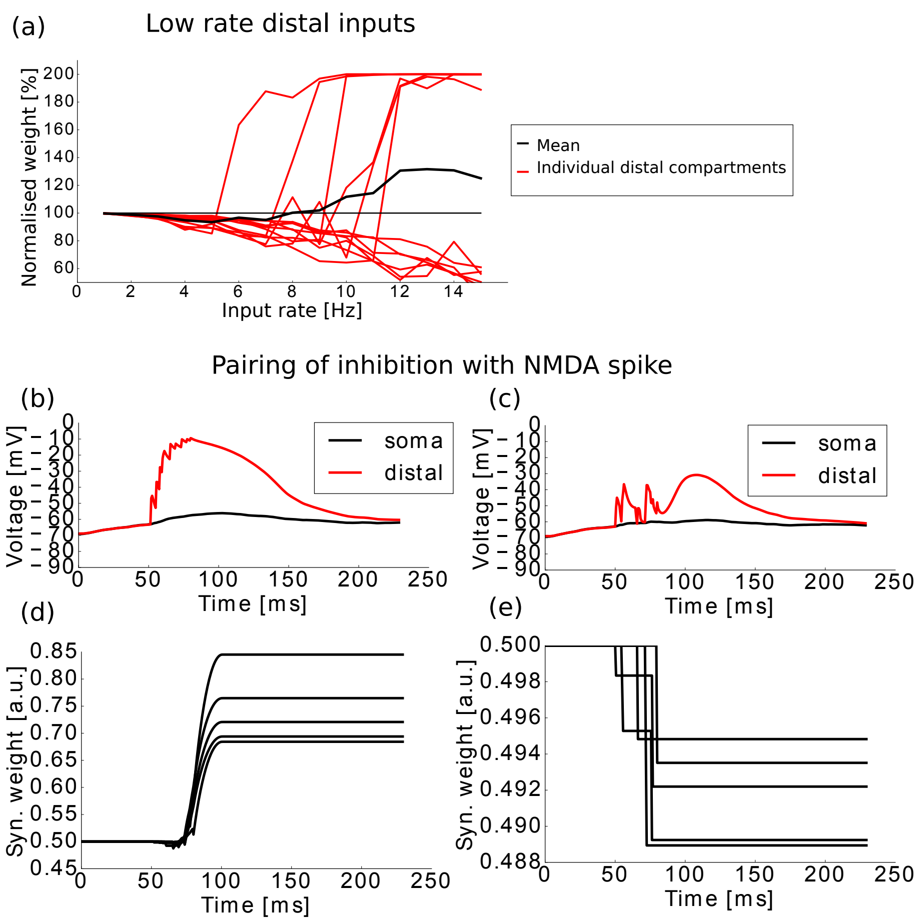

Supplementary Figure 1 Depression at distal compartments.
(**a**) The same simulation as in Figure 2f is repeated, but for all rates between 1 and 15Hz and with steps of 1Hz. The traces for all individual distal compartments shown in Figure 1a are plotted (red), and reveal synaptic depression at lower activation rates than those leading to potentiation. The mean over all compartments (black) somewhat obscures this fact, due to the strong potentiation compared to depression. (**b,d**) Five synapses connected distally evoke an NMDA spike (b), leading to synaptic potentiation (d). (**c,e**) Using the same activation as in panel (b) with the addition of five inhibitory synapses fails to evoke an NMDA spike (c), leading to synaptic depression (e).

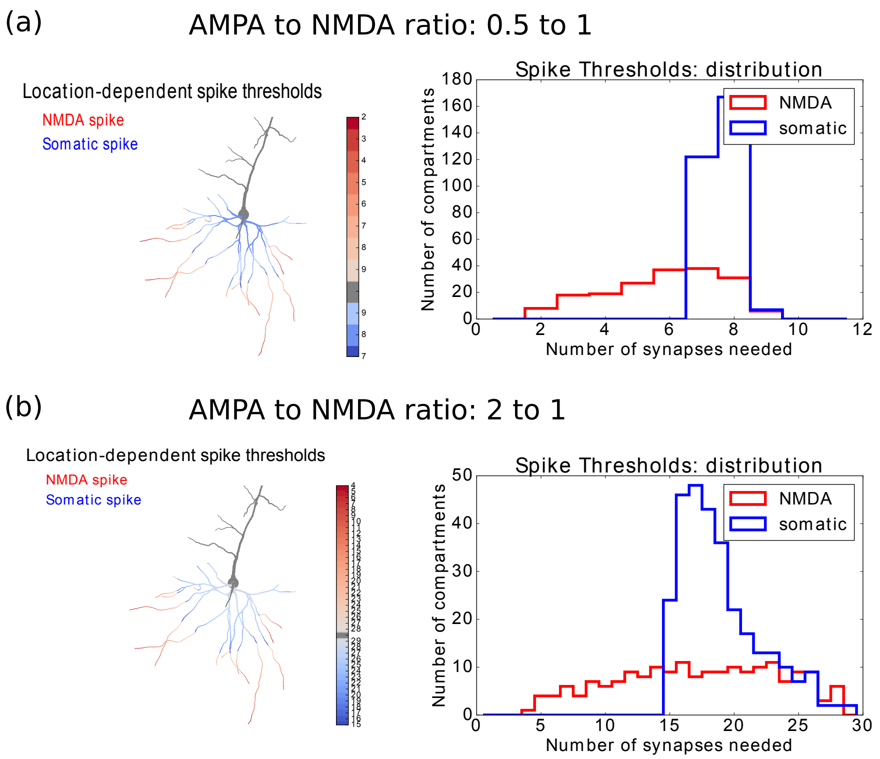

Supplementary Figure 2 Thresholds for somatic and NMDA spikes for different AMPA/NMDA ratios.

(**a**) Analogous to Figures 1c and d, but with an AMPA to NMDA ratio of 0.5. (**b**) Analogous to Figures 1c and d, but with an AMPA to NMDA ratio of 2.

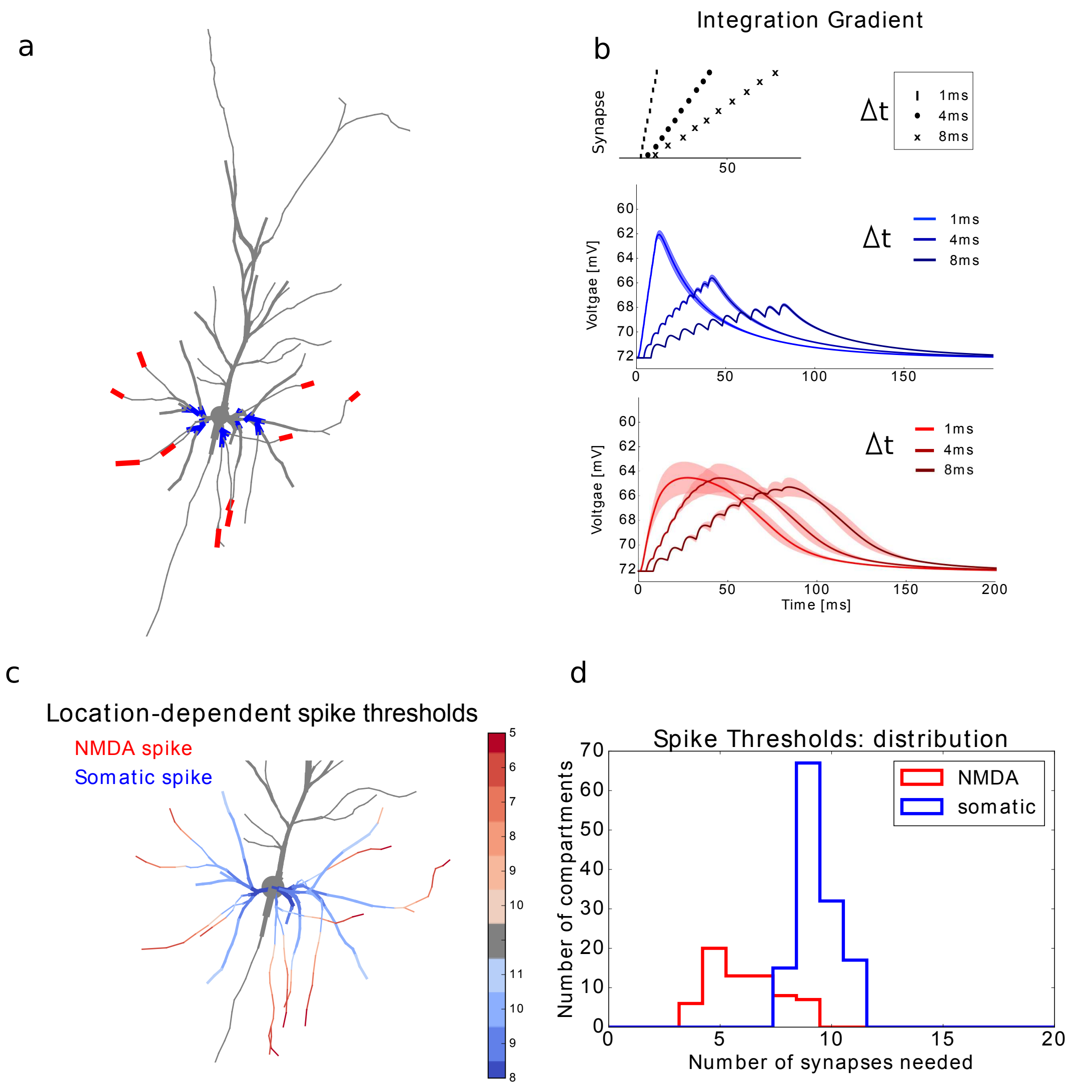

Supplementary Figure 3 Layer 2/3 pyramidal neuron: location dependence of local and global regenerative events.
(See Figure 1, but now for a layer 2/3 neuron morphology)

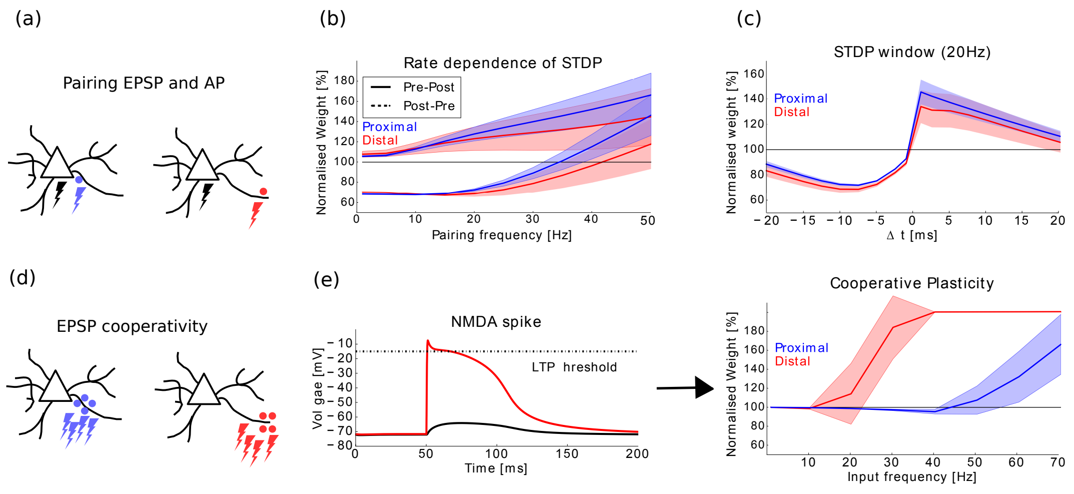

Supplementary Figure 4 Layer 2/3 pyramidal neuron: plasticity gradient along basal dendrites. (See Figure 2, but now for a layer 2/3 neuron morphology)

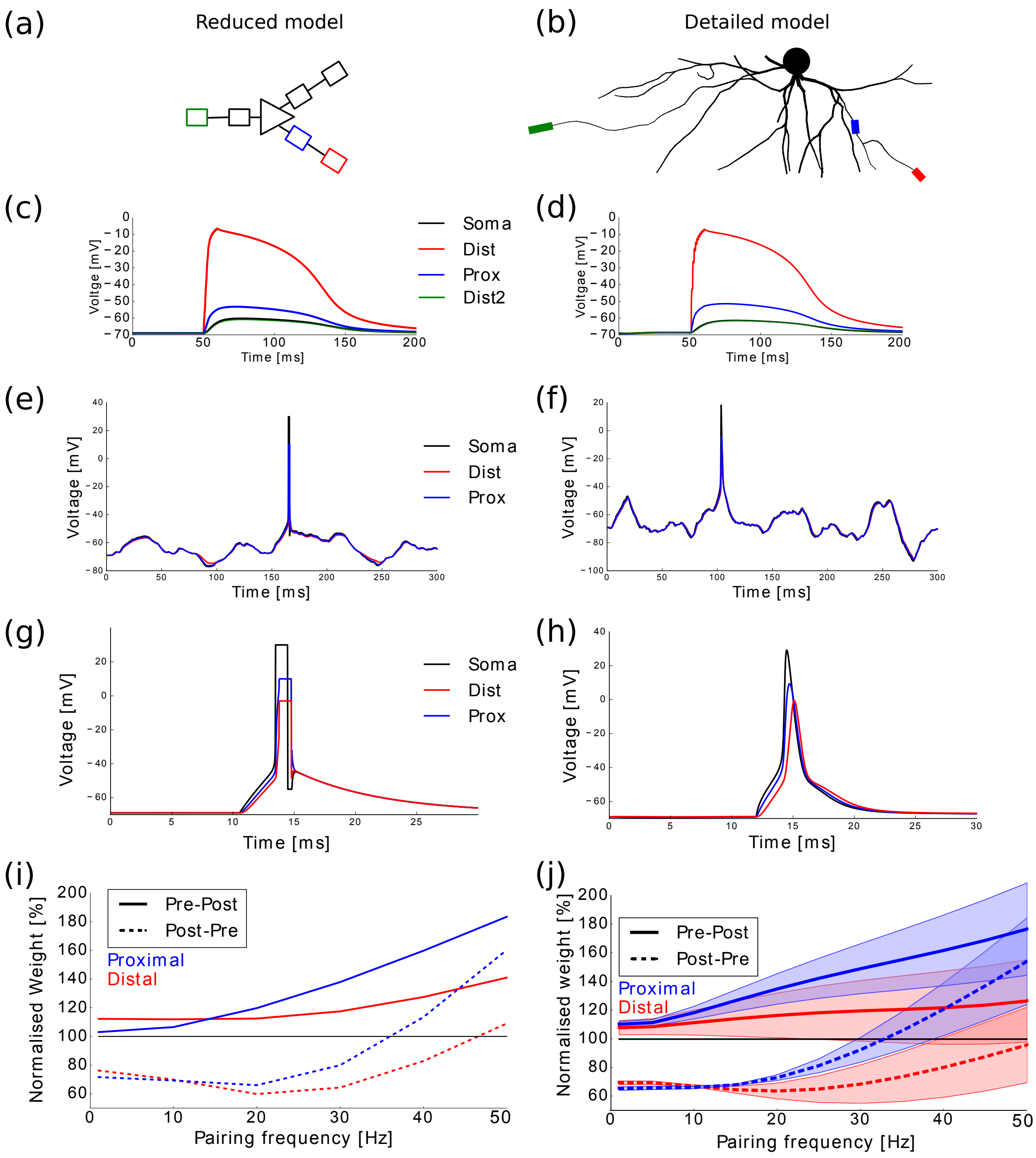

Supplementary Figure 5 Comparison of the reduced and the detailed model.
(**a,b**) Dendrites in the reduced model are composed of two compartments, one representing a proximal region and one a distal region of the full model. (**c,d**) Evoking an NMDA spike at a distal compartment (red) will propagate a proximal compartment on the same dendrite (blue), the soma (black) and eventually up to distal compartments in other dendrites (green). (**e,f**) Noisy currents are injected in the soma of both models. Somatic (black), proximal (blue) and distal (red) voltages are shown. (**g,h**) Comparison of the spikes in both models. While in the reduced model all spikes have identical shapes, in the full model the spike can vary somewhat depending on the input strength and firing rate. (**i,j**) Comparison of the plasticity protocol as in Figure 2b gives similar results.

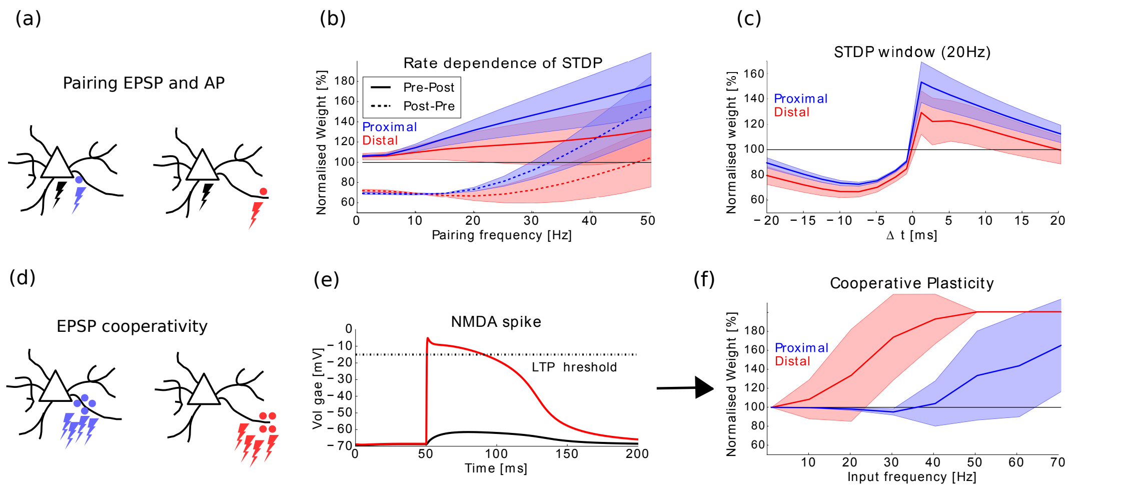

Supplementary Figure 6 Both AMPA and NMDA plastic: Plasticity gradient along basal dendrites. (Analogous to Figure 2, but both the AMPA and NMDA components of the synapses are plastic)

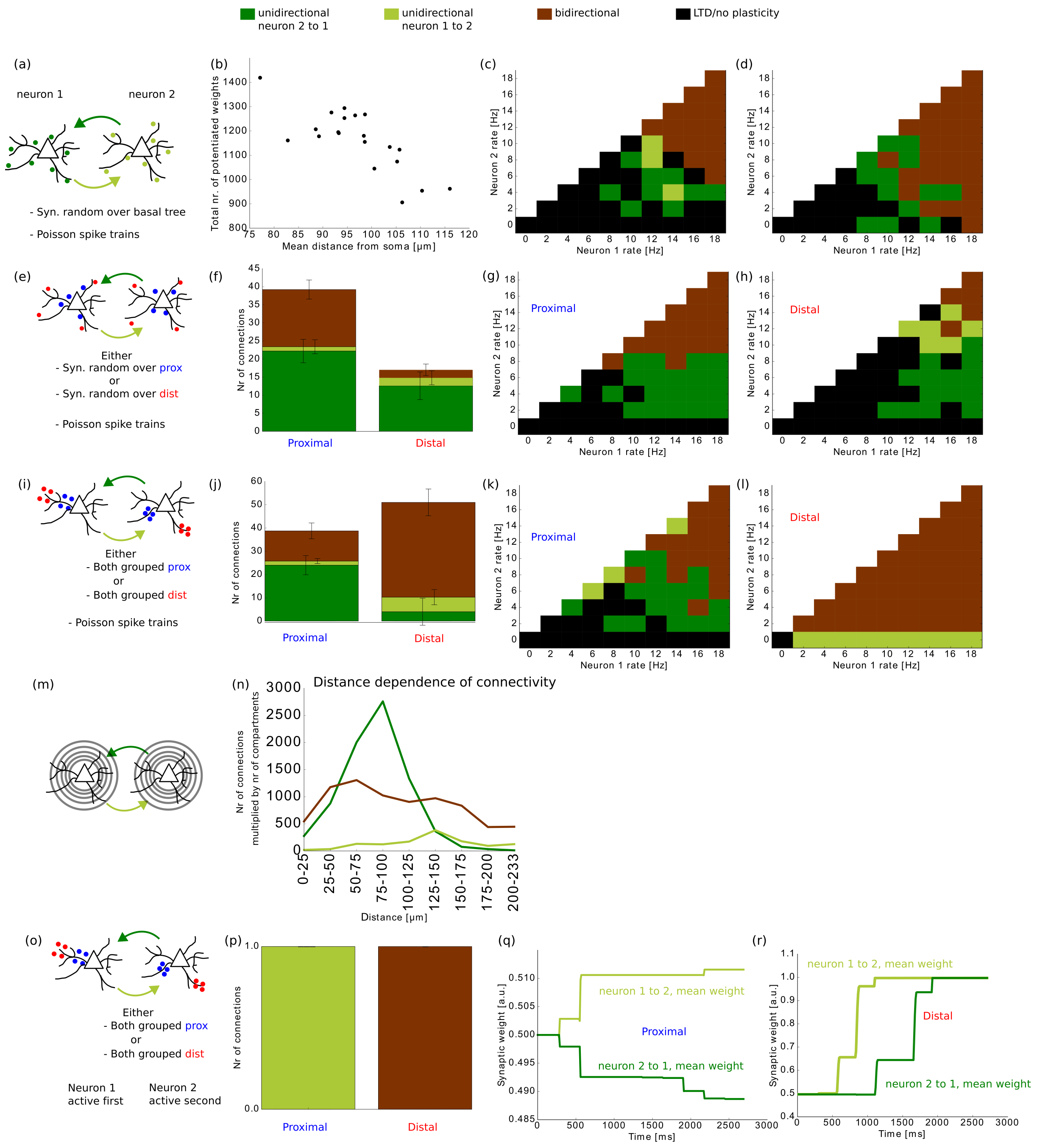

Supplementary Figure 7 Both AMPA and NMDA plastic: Predicted connectivity along dendrites. (Analogous to Figure 3, but both the AMPA and NMDA components of the synapses are plastic)

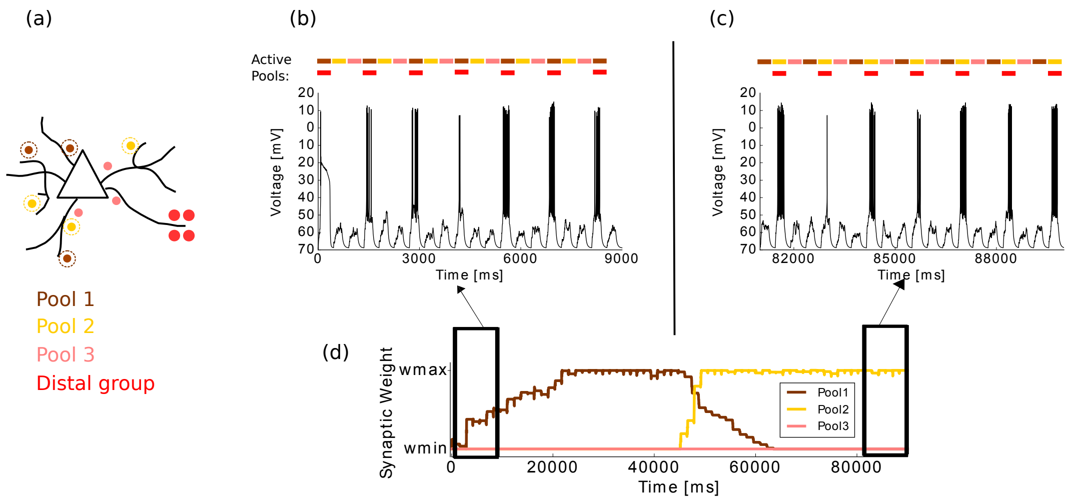

Supplementary Figure 8 Both AMPA and NMDA plastic: Distally evoked plateau potentials can switch the direction of plasticity at other synapses.
(Analogous to Figure 4, but both the AMPA and NMDA components of the synapses are plastic)

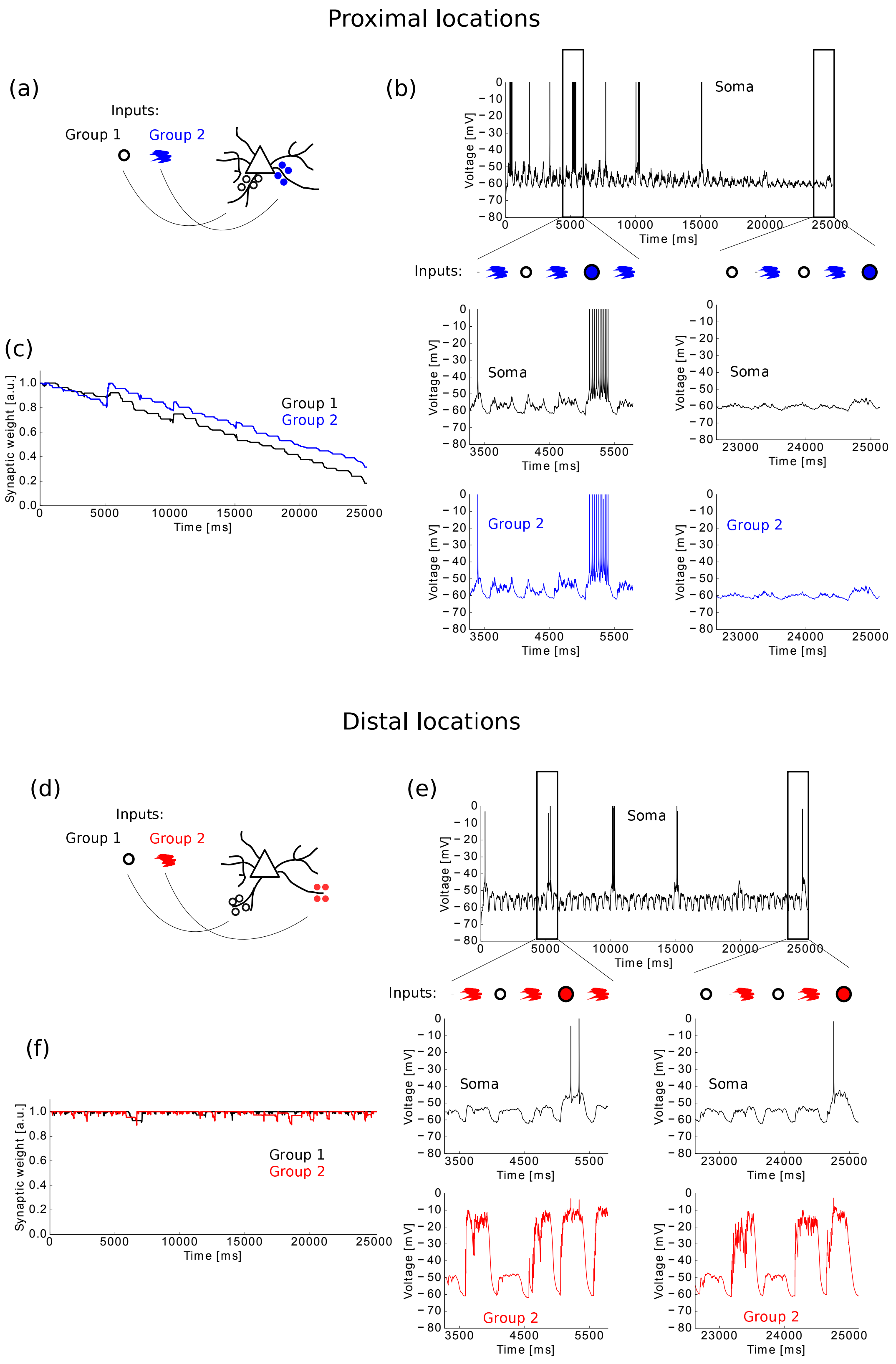

Supplementary Figure 9 Both AMPA and NMDA plastic: Subthreshold inputs are maintained by dLTP.

(Analogous to Figure 5, but both the AMPA and NMDA components of the synapses are plastic)

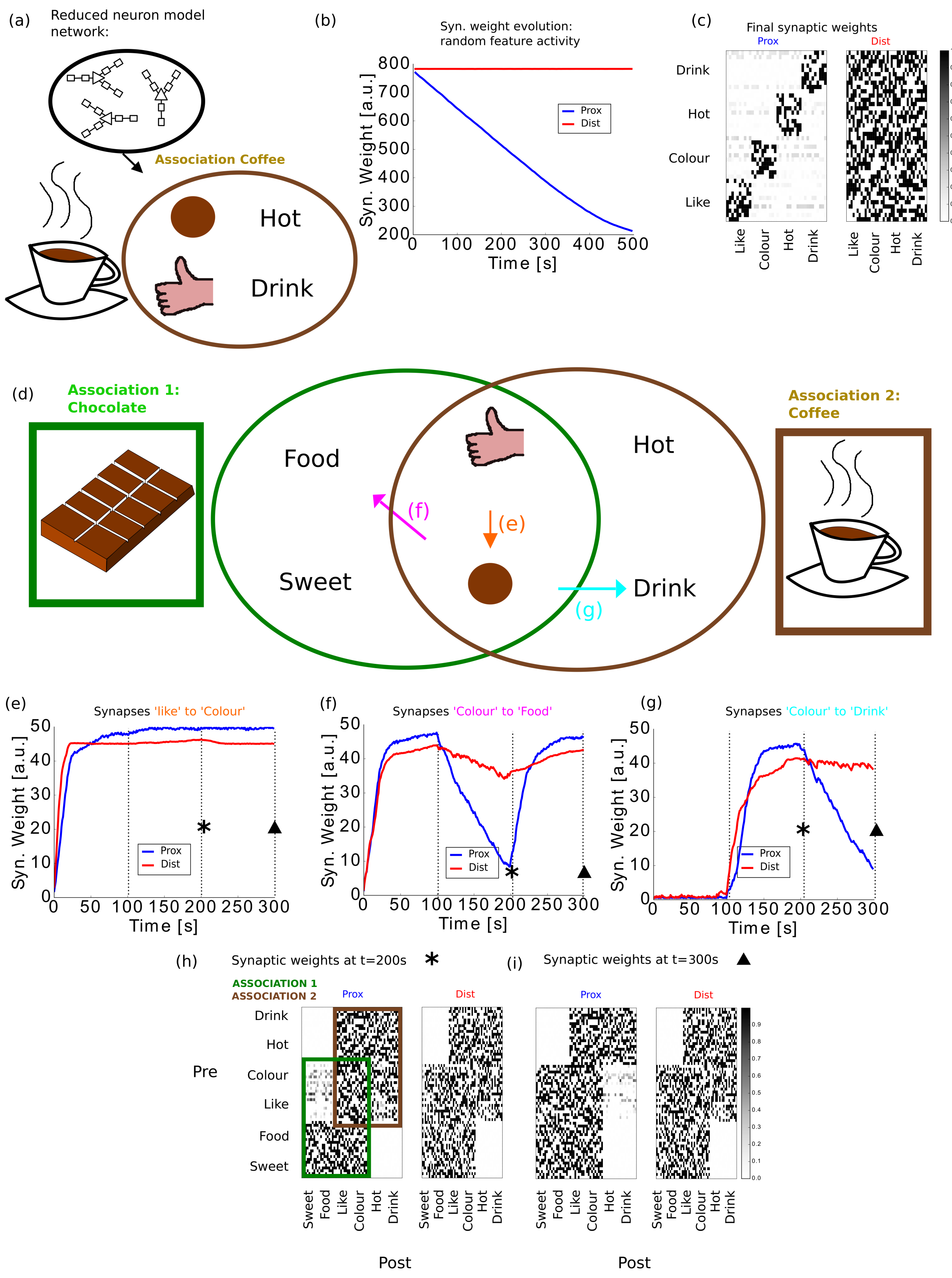

Supplementary Figure 10 Both AMPA and NMDA plastic: In networks, dLTP can protect previously learnt connections.
(Analogous to Figure 6, but both the AMPA and NMDA components of the synapses are plastic)

Supplementary Methods

Supplementary Figure 1a: The distal compartments shown in Figure 1a are connected with 10 synapses each, all with an initial weight of 0.5. The 10 synapses are activated using a Poisson process with the same average rate, lasting 2s. This protocol is repeated with rates between 1Hz to 15Hz with steps of 1Hz. The red lines represent all individual compartments, the black line is the mean over these compartments.

Supplementary Figure 1b-e: Five excitatory and five inhibitory synapses are connected to the same distal compartment. The excitatory synapses have plastic AMPA components ($g_{\mathrm{max}} =$ 1500nS) and fixed NMDA components ($g_{\mathrm{NMDA}} = 1500$nS). The inhibitory synapses are not plastic ($g_{\mathrm{GABA}} = 1000$nS) and an activated inhibitory synapse will result in an instantaneous rise of GABA conductances by an amount of g_{GABA}. This is followed by an exponential decay with a time constant of 10ms. The current flowing through GABA receptors is modelled by

$$I_{\mathrm{GABA}}(t) = g(t) \cdot (u(t) - E_{\mathrm{GABA}})$$

where $g(t)$ is the instantaneous GABA conductance and $E_{\mathrm{GABA}} = -75$mV is the GABA reversal potential. An additional noisy current with mean 100pA and standard deviation of 10pA is injected in the soma. For each excitatory synapse, a Poisson spiketrain is generated with mean rate of 80Hz and for a duration of 30ms. In panel (b), only excitatory synapses are activated, leading to the weight changes in panel (d). In panel (c), each spiketrain activating an excitatory synapse simultaneously activates an inhibitory synapse. The inhibition prevents the induction of an NMDA spike, leading to weight changes in panel (e).

Supplementary Figure 2: We refer to the methods of Figures 2c,d, with the following changes: for panel a the NMDA conductance is 3000pS and for panel b the NMDA conductance is 750pS. The AMPA conductance remains 1500pS for all simulations.

Supplementary Figures 3 and 4: We refer to the methods for Figure 1 and 2, with the only difference that the analysis is now performed in a layer 2/3 morphology and the axonal sodium channel densities are: for the initial two segments, 8000 and 7000 pS μm^{-2}, for the rest of the axon, 5000 pS μm^{-2}.

Supplementary Figure 5: In panels (c,d), The same protocol as in Figure 2e was repeated for both the reduced and the detailed model. The voltage traces for the compartments shown in (a,b) are stored.
In panels (e,f), a noisy current is injected in the soma of the reduced model and the detailed model. For the reduced model, we used a mean of 200pA and a standard deviation of 2500pA. For the detailed model, we used a mean of 100pA and a standard deviation of 300pA. We remind the reader that these values, especially in the reduced model, are abstract parameters and do not necessarily represent realistic values, but were chosen in order to reproduce a certain behaviour. In panels (g,h), a step current of 3000pA was injected in the soma of our reduced model during 3ms. A step current of 1000pA was injected during 3ms in the soma of our full model. No other stimulation was used and the membrane voltage in the soma, proximal (blue) and distal (red) compartments as shown in (a,b) were stored.
In panel (i), the same protocol as in Figure 2b was reproduced in our reduced model. Figure 2b is shown as panel (j) for reference.

Supplementary Figures 6 to 10: We refer to the methods of Figures 2 to 6, with the following differences:

The simulations in the main text were performed with a plastic AMPA component and a fixed NMDA component of synapses. Therefore, the NMDA component was able to contribute to a neuron's depolarisation, even when the AMPA component reached its minimum weight. However, experimental results have shown that long-lasting changes in neuronal activity maintain the ratio of AMPA to NMDA across a neuron [1]. To account for this plasticity of synaptic NMDA receptors, we performed simulations with both AMPA and NMDA component plastic. These gave qualitatively similar results.

The axonal sodium channel densities are: for the initial two segments, 8000 and 7000 $\mathrm{pS\,\mu m^{-2}}$, for the rest of the axon, 5000 $\mathrm{pS\,\mu m^{-2}}$.

The maximal AMPA conductance is 1.5nS and the maximal NMDA conductance is 3nS.

Supplementary Figure 7a-d: 20 synaptic connections are made instead of 15.

Supplementary Figure 8: the maximum weight is reduced to 0.65, the minimum weight is increased to 0.55.

Supplementary Figure 9: the coloured noisy current has standard deviation $\sigma_{\mathrm{noise}} = 10\mathrm{pA}$, mean $\mu_{\mathrm{noise}} = 115\mathrm{pA}$. An activation event consists of Poisson-distributed spikes at an average rate of 30Hz at the member synapses during 350ms. Two subsequent activations are separated by a 150ms interval. Every 10 activations (i.e. 5 for each pool), both pools are activated simultaneously.

Supplementary Figure 10: the parameter A_{inhib} has the value 750pS for panels 10b-c and 600pS for panels 10e-i

References

[1] a J Watt, M C van Rossum, K M MacLeod, S B Nelson, and G G Turrigiano. Activity coregulates quantal AMPA and NMDA currents at neocortical synapses. *Neuron*, 26(3):659–670, 2000.
